# Comparative Effectiveness of Anticoagulants in Patients With Cancer-Associated Thrombosis

**DOI:** 10.1001/jamanetworkopen.2023.25283

**Published:** 2023-07-24

**Authors:** Irbaz Bin Riaz, Harry Fuentes, Yihong Deng, Syed Arsalan Ahmed Naqvi, Xiaoxi Yao, Lindsey R. Sangaralingham, Damon E. Houghton, Leslie J. Padrnos, Fadi E. Shamoun, Waldemar E. Wysokinski, Robert D. McBane

**Affiliations:** 1 Department of Hematology and Oncology, Mayo Clinic, Phoenix, Arizona; 2 Department of Hematology and Oncology, Mayo Clinic, Rochester, Minnesota; 3 Robert D. and Patricia E. Kern Center for the Science of Health Care Delivery, Mayo Clinic, Rochester, Minnesota; 4 Division of Health Care Delivery Research, Robert D. and Patricia E. Kern Center for the Science of Health Care Delivery, Mayo Clinic, Rochester, Minnesota; 5 Gonda Vascular Center, Mayo Clinic, Rochester, Minnesota; 6 Department of Cardiovascular Medicine, Mayo Clinic, Rochester, Minnesota; 7 Department of Cardiovascular Medicine, Mayo Clinic, Phoenix, Arizona

## Abstract

**Importance:**

Patterns of clinical utilization and comparative effectiveness of anticoagulants for cancer-associated thrombosis (CAT) remain largely unexplored.

**Objectives:**

To assess patterns of and factors associated with anticoagulant use and to evaluate the comparative effectiveness of contemporary anticoagulants in patients with active cancer in a clinical setting.

**Design, Setting, and Participants:**

This retrospective cohort study obtained deidentified OptumLabs electronic health record claims data from January 1, 2012, to September 30, 2019. Adult patients (≥18 years of age) with a primary cancer diagnosis (except skin cancer) during at least 1 inpatient or 2 outpatient visits within 6 months before the venous thromboembolism (VTE) date were included. Data were analyzed from April 2020 to September 2021.

**Exposures:**

The patients were grouped according to the anticoagulant prescribed: (1) direct oral anticoagulants (DOACs), (2) low-molecular-weight heparin (LMWH), and (3) warfarin.

**Main Outcomes and Measures:**

Odds ratios (ORs) were used to present the association between factors of interest and utilization of anticoagulants. Main efficacy outcomes included risk of VTE recurrence and all-cause mortality. Main safety outcomes included the risk of hospitalization due to major bleeding. Relative treatment effect estimates were expressed as hazard ratios (HRs) with 95% CIs.

**Results:**

This study included 5100 patients (mean [SD] age, 66.3 [12.3] years; 2670 [52.4%] women; 799 [15.7%] Black, 389 [7.6%] Hispanic, and 3559 [69.8%] White individuals). Overall, 2512 (49.3%), 1488 (29.2%), and 1460 (28.6%) filled prescriptions for DOACs, LMWH, and warfarin, respectively. The median (IQR) treatment duration was 3.2 (1.0-6.5) months for DOACs, 3.1 (1.0-6.8) months for warfarin, and 1.8 (0.9-3.8) months for LWMH. Patients with lung (OR, 2.07; 95% CI, 1.12-3.65), urological (OR, 1.94; 95% CI,1.08-3.49), gynecological (OR, 4.25; 95% CI, 2.31-7.82), and colorectal (OR, 2.26; 95% CI, 1.20-4.32) cancer were associated with increased prescriptions for LMWH compared with DOACs. LMWH (HR, 1.47; 95% CI, 1.14-1.90) and warfarin (HR, 1.46; 95% CI, 1.13-1.87) were associated with an increased risk of VTE recurrences compared with DOACs. LMWH was associated with an increased risk of major bleeding (HR, 2.27; 95% CI, 1.62-3.20) and higher all-cause mortality (HR, 1.61; 95% CI, 1.15-2.25) compared with DOACs.

**Conclusions and Relevance:**

In this comparative effectiveness study of claims-based data, patients with CAT received anticoagulation for a remarkably short duration in clinical settings. DOACs was associated with a lower risk of VTE recurrence, major bleeding, and mortality. Warfarin may still be considered for patients with contraindications to DOACs and those with poor persistence on LMWH.

## Key Points

**Question:**

What are the patterns of anticoagulant utilization and the anticoagulants associated with the lowest risk for venous thromboembolism (VTE) recurrence in patients with cancer in a clinical setting?

**Findings:**

In this comparative effectiveness study with 5100 adult patients, twice as many patients were prescribed direct oral anticoagulants (DOACs) as other classes, and cancer type was associated with the choice of anticoagulant prescription. Use of DOACs was associated with a 50% risk reduction in VTE recurrence compared with low-molecular-weight heparin (LMWH) and warfarin and a 60% risk reduction in all-cause mortality compared with LMWH; DOACs were also associated with reduced risk of major bleeding and gastrointestinal tract bleeding compared with LMWH.

**Meaning:**

In this study, DOACs were associated with a higher persistence rate, lower risk of VTE recurrence, lower risk of major bleeding, and improved mortality.

## Teaser

This comparative effectiveness research assesses patterns of and factors associated with anticoagulant use and evaluates the risk of venous thromboembolism among patients with active cancer.

## Introduction

Management of cancer-associated thrombosis (CAT) is complicated owing to several variables, including cancer-specific thrombotic and bleeding risk, cancer treatment–associated complications, frequent invasive procedures, and constitutional adverse effects such as nausea, vomiting, and anorexia, which may impact medication absorption and adherence. These add to the risk of venous thromboembolism (VTE) recurrence and major bleeding, which both carry high case fatality rates. Treating patients with cancer-associated VTE is therefore challenging due to the delicate balance between these extremes.^1^

For nearly 20 years, guideline recommendations for patients with cancer-associated VTE was low-molecular-weight heparin (LMWH)^2,3,4,5,6^ based on the results of the CLOT trial.^7^ Trials of other LMWH preparations, however, were not able to duplicate these results.^8,9,10,11^ More recently, 4 randomized clinical trials (RCTs) have found that direct oral anticoagulants (DOACs) offer a reasonable alternative to parenteral dalteparin for the acute treatment of cancer-associated VTE with acceptable efficacy and safety outcomes.^12,13,14,15^ As such, DOACs have received guideline endorsement for treating acute VTE in this setting.^16,17^ Despite these findings and guideline statements, warfarin has remained a common treatment strategy for community-based oncology practices for various reasons, including cost and patient preference for oral over parenteral medications.^18,19^ The comparative utilization of these 3 classes of anticoagulant in clinical oncology practices has not been thoroughly explored.

DOACs decrease VTE recurrence and major bleeding as compared with LMWH in clinical trial settings.^13^ Whether DOACs are more effective and safer than LMWH and how they compare with warfarin in a clinical oncology context is not established. Thus, we used claims-based data from OptumLabs to assess the utilization patterns and the comparative efficacy and safety of these available anticoagulant classes.

## Methods

This comparative effectiveness study was reported in accordance with the Professional Society for Health Economics and Outcomes Research (ISPOR) reporting guideline for comparative effectiveness research.^20^ Deidentified administrative claims data from OptumLabs Data Warehouse (OLDW)^21^ were queried to identify patients with active cancer and acute VTE from January 1, 2012, to September 30, 2019. The Mayo Clinic institutional review board exempted this study from review and the requirement for informed consent due to the analysis of preexisting, deidentified data.

### Study Population

Adult patients (≥18 years of age) with a primary cancer diagnosis (except skin cancer) with at least 1 inpatient or 2 outpatient visits within 6 months before the VTE date were included. Patients with hematological malignant neoplasms and solid tumors, including those with lung, urologic or genitourinary [GU], breast, colorectal, gynecological, pancreaticobiliary, upper gastrointestinal [GI], brain, head and neck, and musculoskeletal cancers, were included. Incident VTE was identified using *International Classification Disease* (*ICD*) billing codes between January 1, 2012, and September 30, 2019 (eFigure 1 in Supplement 1). The first diagnosis date of VTE was defined as the date of incident diagnosis. The study cohort was limited to patients who filled an anticoagulant prescription within 30 days after the VTE date. Patients were then categorized into 1 of 3 groups ([1] DOAC, [2] LMWH, or [3] warfarin) based on the initial prescription filled. The first fill date of a specific anticoagulant was defined as the index therapy and treatment date. Study drug discontinuation was defined as not refilling a medication after 30 days of the end of last treatment episode, which is calculated based on fill date and supply.

Patients who crossed over to a different anticoagulant within the first year were excluded from the analysis. Patients were also excluded from the analysis for any of the following reasons: (1) crossed over to a different anticoagulant within the first year; (2) prior history of VTE; (3) filled prescription for an oral anticoagulant (warfarin and DOAC) less than 1 year prior to the VTE index date; or (4) less than 1 year of continuous insurance coverage prior to the VTE index date. Eligible individuals with missing data were removed and were not included in statistical analyses. Detailed inclusion and exclusion criteria are outlined in the eMethods in Supplement 1.

### Follow-Up

Follow-up originated at the VTE index date and continued until the end of treatment. This was defined as (1) date of index anticoagulant discontinuation; (2) end of enrollment in health insurance plan; (3) 1 year after VTE index date; (4) end of the study period (September 30, 2019); or (5) date of patient death.

### Outcomes of Interest

The main efficacy end points included any VTE recurrence and all-cause mortality. The main safety end points included any episode of major bleeding and sites of bleeding (GI, GU, intracranial bleeding) (eTable 2 in Supplement 1).^22^

### Statistical Analysis

Analyses were performed between April 2020 and September 2021. Baseline characteristics (including but not limited to cancer type, presence of metastatic disease, baseline intervention [chemotherapy and surgery], baseline comorbidities, and Charlson Comorbidity Index) of the treatment cohorts were reported. Multinomial logistic regression was used to assess factors associated with use of DOAC relative to other anticoagulants (LMWH and warfarin) and presented as odds ratios (ORs) and 95% CIs. Kaplan-Meier curves were plotted to assess the differences in time to medication discontinuation among the 3 groups.

Propensity score (PS) with inverse probability of treatment weighting was used to balance differences in baseline characteristics among the 3 treatment groups.^23^ All baseline characteristics listed were included in the PS models to derive the PS and the average treatment effect weights (Table 1; eTable 3 in Supplement 1). The standardized mean difference was used to assess the balance of covariates, and a standardized mean difference less than 0.1 was considered acceptable.

**Table 1.** Baseline Sociodemographic and Clinical Characteristics of Patients Included in the Study

Characteristic	Patients, No. (%)			
	DOAC (n = 2152)	LMWH (n = 1488)	Warfarin (n = 1460)	Total (N = 5100)
Age, mean (SD), y	67.4 (12.1)	63.4 (12.8)	67.6 (11.6)	66.3 (12.3)
Gender				
Women	1129 (52.5)	792 (53.2)	749 (51.3)	2670 (52.4)
Men	1023 (47.5)	696 (46.8)	711 (48.7)	2430 (47.6)
Race				
Asian	48 (2.2)	36 (2.4)	23 (1.6)	107 (2.1)
Black	350 (16.3)	211 (14.2)	238 (16.3)	799 (15.7)
Hispanic	168 (7.8)	127 (8.5)	94 (6.4)	389 (7.6)
White	1458 (67.8)	1053 (70.8)	1048 (71.8)	3559 (69.8)
Unknown	128 (5.9)	61 (4.1)	57 (3.9)	246 (4.8)
Cancer type				
Lung	351 (16.3)	285 (19.2)	277 (19.0)	913 (17.9)
Urologic	375 (17.4)	167 (11.2)	288 (19.7)	830 (16.3)
Breast	352 (16.4)	144 (9.7)	203 (13.9)	699 (13.7)
Colorectal	251 (11.7)	138 (9.3)	191 (13.1)	580 (11.4)
Hematological	262 (12.2)	125 (8.4)	149 (10.2)	536 (10.5)
Gynecological	132 (6.1)	170 (11.4)	107 (7.3)	409 (8.0)
Pancreaticobiliary	127 (5.9)	188 (12.6)	86 (5.9)	401 (7.9)
Upper gastrointestinal	86 (4.0)	118 (7.9)	69 (4.7)	273 (5.4)
Brain	80 (3.7)	96 (6.5)	51 (3.5)	227 (4.5)
ENT	90 (4.2)	48 (3.2)	33 (2.3)	171 (3.4)
Musculoskeletal	32 (1.5)	32 (2.2)	20 (1.4)	84 (1.6)
Cancer of unknown primary	41 (1.9)	20 (1.3)	13 (0.9)	74 (1.5)
Genitourinary	<11 (0.4)^a^	<11 (0.3)^a^	<11 (0.2)^a^	>11 (0.3)^a^
Other	<11 (0.1)^a^	<11 (0.1)^a^	<11 (0.1)^a^	<11 (0.1)^a^
Metastatic solid tumor	1164 (54.1)	1104 (74.2)	795 (54.5)	3063 (60.1)
Baseline chemotherapy within 6 mo before index date	1126 (52.3)	920 (61.8)	741 (50.8)	2787 (54.6)
Surgery within 6 mo before index date	775 (36.0)	557 (37.4)	640 (43.8)	1972 (38.7)
VTE type				
DVT	1033 (48.0)	663 (44.6)	709 (48.6)	2405 (47.2)
PE	953 (44.3)	677 (45.5)	624 (42.7)	2254 (44.2)
DVT and PE	166 (7.7)	148 (9.9)	127 (8.7)	441 (8.6)
VTE visit type				
Hospitalization	1263 (58.7)	1003 (67.4)	1090 (74.7)	3356 (65.8)
ED	709 (32.9)	382 (25.7)	266 (18.2)	1357 (26.6)
Office	180 (8.4)	103 (6.9)	104 (7.1)	387 (7.6)
VTE risk score^b^				
1	1098 (51.0)	556 (37.4)	690 (47.3)	2344 (46.0)
2	683 (31.7)	488 (32.8)	510 (34.9)	1681 (33.0)
3	371 (17.2)	444 (29.8)	260 (17.8)	1075 (21.1)
Charlson Comorbidity Index				
Mean (SD)	7.6 (3.8)	8.6 (3.2)	7.6 (3.7)	7.9 (3.6)

Abbreviations: DOAC, direct oral anticoagulants; DVT, deep vein thrombosis; ED, emergency department; ENT, ear, nose, and throat; LMWH, low-molecular-weight heparin; PE, pulmonary embolism; VTE, venous thromboembolism.

^a^ Number of patients masked to protect confidentiality.

^b^ VTE risk was determined by cancer type (eTable 1 in Supplement 1). Risk score of 1 indicates low risk of VTE; 2, intermediate; and 3, high.

Weighted Cox proportional hazards regression with a robust variance estimator was used to assess outcomes. The event rates per 100 person-years and hazard ratios (HRs) were calculated, and the cumulative incidence curves were plotted. *P* < .05 was considered statistically significant for all 2-sided tests. Sensitivity analyses were also conducted on the cohort with index dates between January 1, 2018, and September 30, 2019, to account for selection bias in the use of different anticoagulant medications.

All analyses were conducted using SAS 9.4 (SAS Institute Inc), R version 4.0.2 (R Foundation for Statistical Computing), and Stata version 14.1 (StataCorp). Detailed methods are available in eMethods in Supplement 1.

## Results

### Population Characteristics

A total of 5100 patients were included (mean [SD] age, 66.3 [12.3] years; 2670 [52.4%] women) as shown in Table 1. Most of the population was represented by White individuals (3559 [69.8%]), with 799 (15.7%) Black and 389 (7.6%) Hispanic participants, from the Southern United States (2218 [43.5%]). Among the different cancer types, the 5 most prevalent were lung (913 [17.9%]), urological (830 [16.3%]), breast (699 [13.7%]), colorectal (580 [11.4%]), and hematologic (536 [10.5%]) cancer. Just over 60% of patients had metastatic disease (3063 [60.1%]) at the time of incident VTE, and 2787 (54.6%) received chemotherapy within the antecedent 6 months. More than one-third of patients (1972 [38.7%]) had also undergone cancer-related surgery within this time interval.

Of the total population, 2512 patients (49.3%) were administered DOACs; 1488 (29.2%), LMWH; and 1460 (28.6%), warfarin. VTE was nearly equally divided into deep vein thrombosis (DVT) (2405 [47.2%]) and pulmonary embolism (PE) (2254 [44.2%]) while 441 (8.6%) had evidence of both DVT and PE at the time of diagnosis. Most cases were diagnosed during hospitalization (3365 [65.8%]) or in the emergency department (ED) (1357 [26.6%]) while a paucity was found during a clinic visit (387 [7.6%]). Additional details of baseline characteristics are provided in eTable 3 in Supplement 1.

### Medication Utilization Patterns

By multinomial regression analysis, younger patients were more likely to be prescribed LMWH (OR per 1-year, 0.97; 95% CI, 0.97-0.98; *P* < .001) (Table 2). Both LMWH and warfarin were more likely to be prescribed for patients with lung, urological, gynecological, and colorectal cancer. LMWH was more likely to be prescribed for patients with musculoskeletal and brain cancer, while warfarin was more likely to be prescribed in patients with breast cancer compared with DOACs.

**Table 2.** Factors Associated With Utilization of Different Medication in Cancer-Associated Thrombosis

Variable and medication^a^	OR (95% CI)^b^	*P* value
**Sociodemographic**		
Age		
LMWH	0.97 (0.97-0.98)	<.001
Race, unknown vs White		
LMWH	0.67 (0.48-0.93)	.02
Warfarin	0.64 (0.46-0.90)	.01
Census region, Northeast vs Midwest		
LMWH	1.56 (1.24-1.96)	<.001
Census region, South vs Midwest		
LMWH	0.74 (0.62-0.88)	<.001
Warfarin	0.56 (0.47-0.66)	<.001
**Cancer type**		
Lung, yes vs no		
LMWH	2.07 (1.18-3.65)	.01
Warfarin	1.87 (1.04-3.37)	.04
Urologic, yes vs no		
LMWH	1.94 (1.08-3.49)	.03
Warfarin	2.04 (1.12-3.73)	.02
Musculoskeletal, yes vs no		
LMWH	2.99 (1.44-6.20)	.003
Brain, yes vs no		
LMWH	2.38 (1.25-4.51)	.01
Upper gastrointestinal, yes vs no		
LMWH	1.92 (1.05-3.50)	.03
Pancreaticobiliary, yes vs no		
LMWH	1.95 (1.08-3.52)	.03
ENT, yes vs no		
LMWH	2.33 (1.18-4.61)	.02
Breast, yes vs no		
Warfarin	1.95 (1.03-3.71)	.04
Gynecological, yes vs no		
LMWH	4.25 (2.31-7.82)	<.001
Warfarin	2.31 (1.22-4.39)	.01
Colorectal, yes vs no		
LMWH	2.29 (1.21-4.32)	.01
Warfarin	2.51 (1.32-4.79)	.01
**Baseline interventions**		
Surgery, yes vs no		
Warfarin	1.25 (1.08-1.45)	.003
Chemotherapy, yes vs no		
LMWH	1.25 (1.07-1.45)	.005
**VTE type**		
DVT vs PE		
Warfarin	1.31 (1.12-1.53)	<.001
**VTE visit type**		
ED vs hospitalization		
LMWH	0.63 (0.53-0.75)	<.001
Warfarin	0.39 (0.32-0.46)	<.001
Office vs hospitalization		
LMWH	0.67 (0.50-0.90)	.01
Warfarin	0.60 (0.45-0.79)	<.001
**VTE risk score**		
2 vs 1		
LMWH	1.64 (1.21-2.22)	.001
3 vs 1		
LMWH	2.87 (1.72-4.80)	<.001
Warfarin	1.77 (1.08-2.89)	.02
**Baseline comorbidities**		
Cardiac arrhythmia, yes vs no		
Warfarin	0.79 (0.66-0.93)	.005
Atrial fibrillation, yes vs no		
LMWH	0.76 (0.58-0.98)	.04

Abbreviations: DVT, deep vein thrombosis; ED, emergency department; ENT, ear, nose, and throat; LMWH, low-molecular-weight heparin; OR, odds ratio; PE, pulmonary embolism; VTE, venous thromboembolism.

^a^ This table outlines the results of multinomial logistic regression. All comparisons were made against direct oral anticoagulants. The ORs with associated 95% CIs reflect the association between 2 groups (for a categorical variable) and for a unit change (in a continuous variable) in patients receiving LMWH or warfarin when compared with DOACs. For example, for cancer type, patients who had lung cancer, compared with those who did not, had increased odds of being prescribed LMWH compared with DOACs.

^b^ Wald 95% confidence limits.

Warfarin was more likely to be prescribed for patients with DVT alone (OR, 1.31; 95% CI, 1.12-1.52; *P* < .001). In contrast, for patients with either combined DVT plus PE or isolated PE, prescribing patterns were similar for LMWH (OR, 1.22; 95% CI, 0.94-1.58; *P* = .13) and warfarin (OR, 1.06; 95% CI, 0.82-1.37; *P* = .66) compared with DOACs.

Patients were more likely to be given DOACs relative to LMWH when treated in the ED (OR, 1.59; 95% CI, 1.33-1.89; *P* < .001) or as an outpatient in the clinics (OR, 1.49; 95% CI, 1.11-2.00; *P* = .01) compared with in-hospital setting. Likewise, DOACs, when compared with warfarin, were more frequently prescribed from either the ED (OR, 2.56; 95% CI, 2.17-3.13; *P* < .001) or outpatient office setting (OR, 1.67; 95% CI, 1.28-2.22; *P* < .001) relative to an in-hospital stay. If patients had a prior history of cardiac arrhythmia as a baseline comorbid condition, they were more likely to be prescribed DOACs compared with warfarin (OR, 1.28; 95% CI, 1.09-1.52; *P* = .005). No statistically significant differences were observed among patients with other baseline comorbidities in terms of being prescribed a medication. The detailed results of multinominal regression are outlined in Table 2 and eTable 4 in Supplement 1. The results of sensitivity analyses were consistent and are provided in eTable 6 in Supplement 1.

### Follow-Up Time

**Figure 1.**
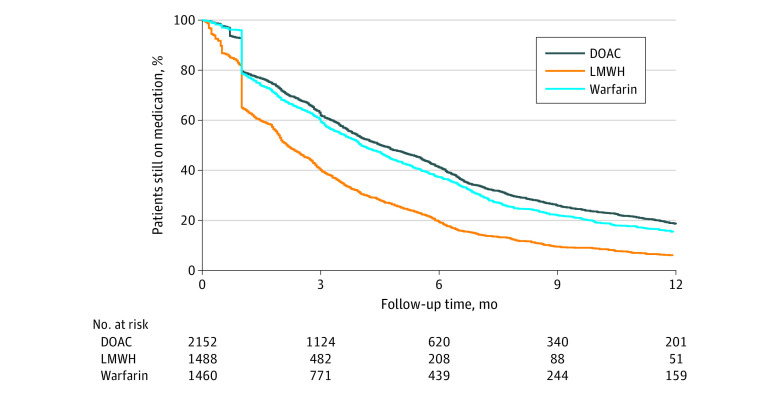
Kaplan-Meier Curves for Patients Still Receiving Medication Over Follow-Up Duration Study drug discontinuation was defined as not refilling a medication after 30 days of the end of last treatment episode, which was calculated based on fill date and supply. The 1-month drop observed here is likely due to the allowed 30-day gap. DOAC indicates direct oral anticoagulant; LMWH, low-molecular-weight heparin.

The median (IQR) treatment duration was 3.2 (1.0-6.5) months for DOACs, 3.1 (1.0-6.8) months for warfarin, and 1.8 (0.9-3.8) months for LWMH (*P* < .001). (Figure 1). At 6 months, a greater percentage of patients continued taking either a DOAC (620 of 2152 [28.8%]) or warfarin (439 of 1460 [30.0%]) compared with LMWH (208 of 1488 [13.9%]).

### Medication Comparative Effectiveness in Weighted Cohorts

**Figure 2.**
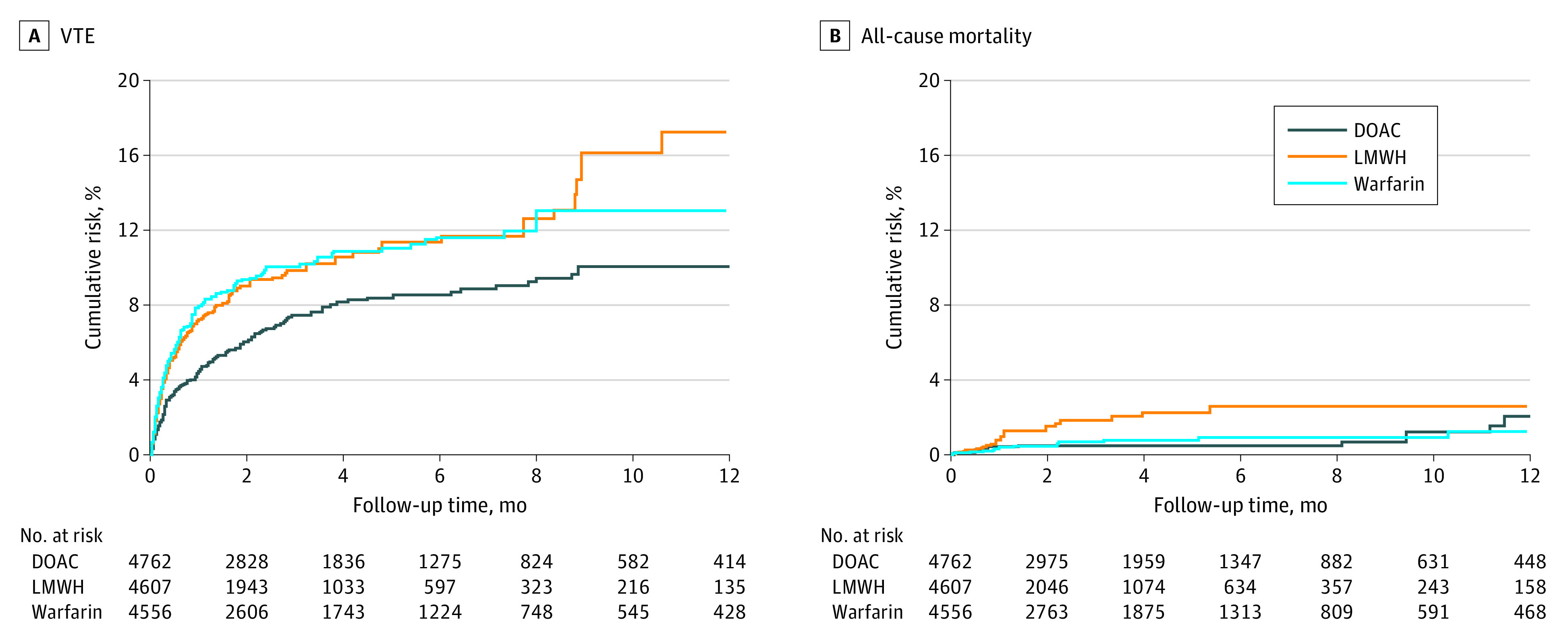
Cumulative Incidence Curves for Venous Thromboembolism (VTE) Recurrence and All-Cause Mortality DOAC indicates direct oral anticoagulant; LMWH, low-molecular-weight heparin.

The results for comparative effectiveness in the weighted cohort are highlighted in Table 3. The 3 groups were balanced after propensity score weighting with SMDs less than 0.1 in all covariates (eTable 5 in Supplement 1). In the weighted cohort, patients receiving LMWH or warfarin were associated with an increased risk of VTE recurrence compared with those prescribed DOACs (LMWH: 39.76 per 100 person-years; warfarin: 29.89 per 100 person-years; DOACs: 20.62 per 100 person-years; LMWH vs DOAC: HR, 1.47; 95% CI, 1.14-1.90; warfarin vs DOAC: HR, 1.46; 95% CI, 1.13-1.87), as shown in Figure 2A. Patients receiving LMWH were associated with an increased risk of all-cause mortality compared with those prescribed DOACs (LMWH: 21.18 per 100 person-years; DOACs: 11.36 per 100 person-years; LMWH vs DOACs: HR, 1.61; 95% CI, 1.15-2.25). In contrast, mortality rates with warfarin did not differ significantly from DOACs (HR, 1.19; 95% CI, 0.85-1.68), as shown in Figure 2B.

**Table 3.** Efficacy and Safety Outcomes in Weighted Cohorts

Outcomes	Patients, No.	Events, No.	Person-years	Events per 100 person-years	Hazard ratio (95% CI)	*P* value
**VTE recurrence**						
LMWH	4607	398	1002.13	39.76	1.47 (1.14-1.90)	.003
Warfarin	4556	456	1526.48	29.89	1.46 (1.13-1.87)	.003
DOAC	4762	332	1609.44	20.62	1 [Reference]	NA
**All-cause mortality**						
LMWH	4607	225	1060.8	21.18	1.61 (1.15-2.25)	.005
Warfarin	4556	221	1627.76	13.59	1.19 (0.85-1.68)	.31
DOAC	4762	193	1696.33	11.36	1 [Reference]	NA
**Hospitalization for major bleeding**						
LMWH	4607	277	1035.84	26.73	2.27 (1.62-3.20)	<.001
Warfarin	4556	179	1609.61	11.1	1.12 (0.78-1.61)	.53
DOAC	4762	166	1681.52	9.88	1 [Reference]	NA
**GI bleeding**						
LMWH	4607	152	1041.21	14.64	1.72 (1.12-2.62)	.01
Warfarin	4556	119	1615.82	7.38	1.03 (0.67-1.59)	.89
DOAC	4762	121	1685.12	7.17	1 [Reference]	NA
**Intracranial bleeding**						
LMWH	4607	62	1058.49	5.88	2.72 (1.24-5.97)	.01
Warfarin	4556	31	1625.72	1.93	1.04 (0.45-2.45)	.92
DOAC	4762	31	1694.92	1.84	1 [Reference]	NA

Abbreviations: DOAC, direct oral anticoagulants; GI, gastrointestinal; LMWH: low-molecular-weight heparin; NA, not applicable; VTE, venous thromboembolism.

Patients receiving LMWH were associated with an increased risk of hospitalizations for major bleeding compared with those prescribed DOACs (LMWH: 26.73 per 100 person-years; DOACs: 9.88 per 100 person-years; LMWH vs DOAC: HR, 2.27; 95% CI, 1.62-3.20) as shown in eFigure 2A in Supplement 1. Many of these events were accounted for by GI bleeding with LMWH compared with DOACs (LMWH: 14.64 per 100 person-years; DOACs: 7.17 per 100 person years; LMWH vs DOAC: HR, 1.72; 95% CI, 1.12-2.62) as shown in eFigure 2B in Supplement 1. The total number of major GU bleeding events was low (<20), which precluded formal statistical testing. Patients receiving LMWH were associated with an increased risk of intracranial bleeding compared with those prescribed DOACs (LMWH: 5.88 per 100 person-years; DOACs: 1.84 per 100 person-years; LMWH vs DOAC: HR, 2.72; 95% CI, 1.24-5.97) as shown in eFigure 2C in Supplement 1. The risks of hospitalization for major bleeding, GI bleeding, and intracranial bleeding in patients receiving warfarin (major bleeding: 11.10 per 100 person-years; GI bleeding: 7.38 per 100 person-years; intracranial bleeding: 1.93 per 100 person-years) were similar to DOACs (major bleeding: HR, 1.12; 95% CI, 0.78-1.61; GI bleeding: HR, 1.03; 95% CI, 0.67-1.59; intracranial bleeding: HR, 1.04; 95% CI, 0.45-2.45).

Sample size in the sensitivity cohort (with index date January 1, 2018, to September 30, 2019) was small and precluded any meaningful statistics. Additionally, post hoc sensitivity analysis for GI bleeding in upper GI malignant neoplasms showed no significant differences among anticoagulants (eTable 7 in Supplement 1).

### Reconciliation With Prior Clinical Data

A literature search was conducted to identify similar studies and to reconcile the findings of our study with the results of those studies. Findings are shown in eFigure 3 in Supplement 1.

## Discussion

Consistent with the general theme of recent RCTs, results from this claims-based cohort of more than 5000 patients include a general preference for DOAC therapy use, with nearly twice as many patients receiving this class of medication compared with other classes. Furthermore, these data reinforce the general efficacy and safety of DOACs in this patient population; they were associated with a nearly 50% reduction in VTE recurrence rates and a more than 2-fold reduction in hospitalization for major bleeding compared with LMWH therapy. The odds of GI and intracranial bleeding were likewise reduced among patients receiving DOACs. Not seen in clinical trials, these data showed an association between DOAC therapy and a significant 60% reduction in all-cause mortality rates relative to LMWH. As such, it is anticipated that these data will help facilitate shared decision-making and inform clinical guidelines for the treatment of such patients.

DOACs have emerged as the most prescribed anticoagulant choice for management of cancer-associated VTE.^24^ Half of patients in this cohort were treated with a DOAC and less than one-third received LMWH (29.1%) despite contemporary guideline recommendations.^2,5,6^ The timeframe of this study (2012-2019) antedated guideline changes endorsing DOAC use in this context.^2,3^ However, DOAC popularity might be attributed to pharmacologic benefits including rapid onset of action, convenient oral administration and dosing, short half-life, lack of monitoring, few drug or food interactions, and good bioavailability.^25,26^ Compared with LMWH, patient preference for oral apixaban, for example, resulted in fewer discontinuations in a recent RCT of cancer-associated acute VTE treatment.^13^ By comparison, warfarin was the least frequently prescribed anticoagulant in this cohort (28.6%). While some studies have shown a decreasing use of warfarin, others have not. In some community oncology practices, nearly half of patients with cancer and acute VTE are still treated with warfarin.^18,19,24^ Guideline recommendations preferring LMWH, specifically dalteparin, over warfarin are based on a single trial.^7^ Trials of other LMWH preparations have not shown an advantage over warfarin.^8,9,10,11^ Warfarin was associated with improved overall survival compared with LMWH in a recent study assessing 9706 propensity score–matched patients with cancer and VTE.^27^ Despite a clear benefit for DOACs over LMWH in the current study, neither major bleeding nor survival outcomes favored DOACs over warfarin; VTE recurrence rates were lower with DOACs.

This study revealed low persistence rates of patients with cancer receiving anticoagulant therapy, with a minority continuing treatment beyond 3 months, similar to findings in another analysis of Medicare data.^27^ Conversely, Guo et al^28^ observed that 87.8% of patients continued to receive DOAC therapy at 3 months. Papakotoulas et al^29^ observed that, on average, patients continued DOAC therapy for 6.8 months. Cohen et al^30^ reported persistence rates at 6 months of 60.6% for DOACs, 38.9% for LMWH, and 51.0% for warfarin. However, lack of crossover between the medications (except for 30-day bridging period of LMWH for warfarin, dabigatran, and edoxaban) could plausibly explain the shorter follow-up period for LMWH in our study. Moreover, different data sources and choice of anticoagulant agents may explain higher adherence rates observed in these studies.

Reasons for treatment discontinuation are complex. It may occur due to tumor-related bleeding complications, GI issues, drug interactions, contraindications due to comorbidities, financial barriers, and concerns about the lack of proven survival benefits.^31,32,33^ These factors contribute to a reduced likelihood of treatment continuation in this patient population.

Anticoagulant utilization patterns appear to vary by regional, sociodemographic, and clinical factors. Younger patients were more likely to receive LMWH. Similarly, for patients being evaluated in the office or ED, DOACs were more frequently prescribed. By comparison, if the clinical interaction was in a hospital setting, DOACs were less likely to be prescribed. These findings are consistent with those of Guo et al,^28^ who found that DOACs were preferred in 40% of outpatients discharged from the hospital, while LMWH was preferred in the inpatient setting. Similarly, DOACs were more frequently prescribed to patients from the South compared with the Midwest, while LMWH use was greater in the Northeast. While we observed no statistically significant differences in prescription patterns by different racial categories, other studies have suggested relatively lower use of DOACs for incident VTE in Black patients, which highlights potential inequity in access to this novel pharmacotherapy.^34,35,36^ The reasons for these different prescribing patterns are not clear, and further exploration of factors associated with anticoagulant choice is required to better understand these differences.

Prescribing patterns also appear to vary by cancer type. DOACs were less likely to be prescribed in patients with lung, urological, colorectal, and breast cancer in our study. Contrasting results were reported with DOACs more likely to be prescribed in patients with prostate and breast cancer.^18^ Varying patient-physician preferences may be responsible for the inconsistency observed between the results, as currently we are lacking evidence to facilitate anticoagulant choice in different cancers. Similarly, in our study, DOACs were more likely to be preferred in patients with concomitant cardiac arrhythmias compared with warfarin, similar to another study.^18^ These preferences are most likely derived from the results of multiple cardiovascular clinical trials that showed superiority of DOACs over warfarin.^37,38,39^ Whether these preferences translate into actual benefit in patients with arrhythmia and cancer is still unknown.

VTE recurrence rates favored DOAC therapy over other anticoagulant choices. DOACs were associated with a 32% risk reduction in VTE recurrence compared with LMWH, consistent with RCTs comparing DOACs with LMWH.^13,14,15^ Cohen et al^30^ found a similar 39% reduction in VTE risk with apixaban compared with LMWH. An important distinction between RCTs and clinical practice is the nearly universal employment of enoxaparin as opposed to dalteparin, which may impact outcomes. Additionally, DOAC trials recruited a low proportion of patients with primary brain tumors and known intracerebral metastases. VTE recurrence rates were also significantly higher for patients receiving warfarin compared with DOACs. While data from RCTs are limited, these findings complement the results of several network meta-analyses that have reported efficacy benefit with DOACs compared with warfarin.^40,41^ Several unanswered questions regarding anticoagulant choice include VTE risk by tumor type, VTE presentation (symptomatic or incidental), and various patient and clinical characteristics.^42,43^

Similarly, patients with cancer receiving anticoagulant therapy are at an increased risk of bleeding, which further hinders anticoagulation delivery and complicates anticoagulant choices. Contrary to guidelines and RCTs (SELECT-D, HOKUSAI-VTE, and CARAVAGGIO), this study found lower rates of major bleeding and GI bleeding with DOACs compared with LMWH in patients with cancer.^12,14,15^ Variable study design, randomization, patient selection, and a relatively higher proportion of patients with upper GI malignant neoplasms included in the trials as opposed to our study could explain the differences. Cohen et al^30^ found a 37% risk reduction in major bleeding with apixaban compared with LMWH but observed no difference for GI bleeding. Furthermore, lower rates of intracranial bleeding likewise favored DOAC use, similar to the results of Cohen et al.^30^ Contrarily, several meta-analyses have suggested increased risk of major bleeding with DOACs compared with LMWH; however, these results are based on limited data and have wide confidence intervals.^40,41,44,45^

Recurrent VTE also presents with an increased risk of mortality, especially in patients with prior history of PE. No anticoagulants to date have been able to achieve overall survival benefit.^7,8,9,10,11,12,13,14,15,44,45^ However, contrary to RCTs, which included near-equal proportion of patients with metastatic disease as our study, we observed lower all-cause mortality with DOACs and an increased risk with LMWH compared with DOACs. These findings indicate that most of these RCTs might have been underpowered to detect such differences. Consistently, another study showed a significant reduction in mortality with rivaroxaban when compared with enoxaparin.^46^ Taken together, these findings reassure and reinforce prior evidence in terms of VTE risk reduction and may be suggestive of lower risk of mortality with DOACs.

The current study has several strengths. We relied on claims data from OLDW, which contain longitudinal health information on enrollees and patients, representing a diverse mixture of ages, races and ethnicities, and geographical regions across the US; conducted multinominal regression analysis to assess factors associated with treatment patterns of utilization; applied propensity score matching for adjustment of differences across baseline sociodemographic and clinical characteristics; assessed comparative effectiveness of DOACs, LMWH, and warfarin using weighted Cox proportional hazard models; and reconciled our design and findings with previous clinical studies to compare differences and consistency between results (eFigure 3 in Supplement 1).

### Limitations

This study has limitations, including information bias (billing inaccuracies and data omissions), the use of *ICD* codes to identify patients with VTE, and the lack of radiological evidence of VTE in the database, which can potentially lead to classification bias for assessment of VTE. The analyses were conducted using US claim-based data so the results could not be extrapolated to other populations. We lacked information on uninsured patients or patients receiving insurance from other federal- or state-regulated insurances; hence, the results may not be representative of such populations. Only observable uncontrolled covariates were accounted for in adjusted multivariate analyses; hence, there is a risk of residual confounding bias. Likewise, this analysis predates the pivotal RCTs; therefore, selection bias in the use of different drugs is likely. The proportion of patients still receiving treatment was used to reflect patient adherence to different medications and assumed that medications supplied were being used, which may not be reflective of true patient adherence. Clinically relevant nonmajor bleeding, which has competing risks among different anticoagulant treatments, was not assessed and may alter the choice of anticoagulant therapy. We assessed all-cause mortality and not VTE or bleeding-specific mortality, which might be more informative to guide the choice of anticoagulant therapy in patients with CAT. We used propensity score matching to address differences in baseline characteristics among the 3 treatment groups. However, it is important to note that there may be additional confounding variables that were not accounted for in outcome assessment. Therefore, careful consideration is warranted when interpreting the results of this study.

## Conclusions

In this study, patients with cancer-associated VTE received anticoagulation therapy for a short duration in clinical practice. The findings suggest that DOACs and warfarin may offer better treatment persistence than LMWH in clinical practice. Warfarin may still be considered for patients with contraindications to DOACs and for those who have poor persistence on LMWH.

## Notes

Supplement 1. **eMethods.** Detailed Methodology **eFigure 1.** Patient Selection Flowchart **eFigure 2.** Cumulative Incidence Curves for (A) Major Bleeding; (B) GI Bleeding; (C) Intracranial Bleeding **eFigure 3.** Reconciliation of Study Characteristics and Results With Previous Clinical Evidence **eTable 1.** VTE Risk Stratification According to CCS Cancer Categories **eTable 2.** *ICD* Codes for Major Bleeding and Different Sites of Bleeding **eTable 3.** Additional Baseline Sociodemographic and Clinical Characteristics of Patients Included in the Study **eTable 4.** Factors Without Significant Associations With Utilization of Anticoagulants in Cancer-Associated Thrombosis **eTable 5.** Sociodemographic and Clinical Characteristics of Patients After Propensity Score Weighting **eTable 6.** Factors Associated With Utilization of Anticoagulants in Sensitivity Cohort of Patients (Index Date: January 1, 2018, to September 30, 2019) With Cancer-Associated Thrombosis **eTable 7.** Post Hoc Sensitivity Analysis for Gastrointestinal (GI) Bleeding in Patients Upper GI Malignant Neoplasm

Supplement 2.
Data Sharing Statement

## References

[1] Xiong W. Current status of treatment of cancer-associated venous thromboembolism. Thromb J. 2021;19(1):21. doi:10.1186/s12959-021-00274-x 33789658 PMC8010277

[2] Farge D, Frere C, Connors JM, ; International Initiative on Thrombosis and Cancer (ITAC) advisory panel. 2019 International clinical practice guidelines for the treatment and prophylaxis of venous thromboembolism in patients with cancer. Lancet Oncol. 2019;20(10):e566-e581. doi:10.1016/S1470-2045(19)30336-5 31492632

[3] Key NS, Khorana AA, Kuderer NM, . Venous thromboembolism prophylaxis and treatment in patients with cancer: ASCO Clinical Practice Guideline Update. J Clin Oncol. 2020;38(5):496-520. doi:10.1200/JCO.19.01461 31381464

[4] Lyman GH, Khorana AA, Falanga A, ; American Society of Clinical Oncology. American Society of Clinical Oncology guideline: recommendations for venous thromboembolism prophylaxis and treatment in patients with cancer. J Clin Oncol. 2007;25(34):5490-5505. doi:10.1200/JCO.2007.14.1283 17968019

[5] Mandalà M, Falanga A, Piccioli A, ; working group AIOM. Venous thromboembolism and cancer: guidelines of the Italian Association of Medical Oncology (AIOM). Crit Rev Oncol Hematol. 2006;59(3):194-204. doi:10.1016/j.critrevonc.2006.05.001 16837209

[6] Wagman LD, Baird MF, Bennett CL, ; National Comprehensive Cancer Network. Venous thromboembolic disease: clinical practice guidelines in oncology. J Natl Compr Canc Netw. 2006;4(9):838-869. doi:10.6004/jnccn.2006.0071 17020664

[7] Lee AYY, Levine MN, Baker RI, ; Randomized Comparison of Low-Molecular-Weight Heparin versus Oral Anticoagulant Therapy for the Prevention of Recurrent Venous Thromboembolism in Patients with Cancer (CLOT) Investigators. Low-molecular-weight heparin versus a coumarin for the prevention of recurrent venous thromboembolism in patients with cancer. N Engl J Med. 2003;349(2):146-153. doi:10.1056/NEJMoa025313 12853587

[8] Deitcher SR, Kessler CM, Merli G, Rigas JR, Lyons RM, Fareed J; ONCENOX Investigators. Secondary prevention of venous thromboembolic events in patients with active cancer: enoxaparin alone versus initial enoxaparin followed by warfarin for a 180-day period. Clin Appl Thromb Hemost. 2006;12(4):389-396. doi:10.1177/1076029606293692 17000884

[9] Hull RD, Pineo GF, Brant RF, ; LITE Trial Investigators. Long-term low-molecular-weight heparin versus usual care in proximal-vein thrombosis patients with cancer. Am J Med. 2006;119(12):1062-1072. doi:10.1016/j.amjmed.2006.02.022 17145251

[10] Lee AYY, Kamphuisen PW, Meyer G, ; CATCH Investigators. Tinzaparin vs warfarin for treatment of acute venous thromboembolism in patients with active cancer: a randomized clinical trial. JAMA. 2015;314(7):677-686. doi:10.1001/jama.2015.9243 26284719

[11] Meyer G, Marjanovic Z, Valcke J, . Comparison of low-molecular-weight heparin and warfarin for the secondary prevention of venous thromboembolism in patients with cancer: a randomized controlled study. Arch Intern Med. 2002;162(15):1729-1735. doi:10.1001/archinte.162.15.1729 12153376

[12] Agnelli G, Becattini C, Meyer G, ; Caravaggio Investigators. Apixaban for the treatment of venous thromboembolism associated with cancer. N Engl J Med. 2020;382(17):1599-1607. doi:10.1056/NEJMoa1915103 32223112

[13] McBane RD II, Wysokinski WE, Le-Rademacher JG, . Apixaban and dalteparin in active malignancy-associated venous thromboembolism: the ADAM VTE trial. J Thromb Haemost. 2020;18(2):411-421. doi:10.1111/jth.14662 31630479

[14] Raskob GE, van Es N, Verhamme P, ; Hokusai VTE Cancer Investigators. Edoxaban for the treatment of cancer-associated venous thromboembolism. N Engl J Med. 2018;378(7):615-624. doi:10.1056/NEJMoa1711948 29231094

[15] Young AM, Marshall A, Thirlwall J, . Comparison of an oral factor Xa inhibitor with low molecular weight heparin in patients with cancer with venous thromboembolism: results of a randomized trial (SELECT-D). J Clin Oncol. 2018;36(20):2017-2023. doi:10.1200/JCO.2018.78.8034 29746227

[16] Lyman GH, Carrier M, Ay C, . American Society of Hematology 2021 guidelines for management of venous thromboembolism: prevention and treatment in patients with cancer. Blood Adv. 2021;5(4):927-974. doi:10.1182/bloodadvances.2020003442 33570602 PMC7903232

[17] Streiff MB, Abutalib SA, Farge D, Murphy M, Connors JM, Piazza G. Update on guidelines for the management of cancer-associated thrombosis. Oncologist. 2021;26(1):e24-e40. doi:10.1002/onco.13596 33275332 PMC7794170

[18] Khorana AA, McCrae KR, Milentijevic D, . Current practice patterns and patient persistence with anticoagulant treatments for cancer-associated thrombosis. Res Pract Thromb Haemost. 2017;1(1):14-22. doi:10.1002/rth2.12002 30046670 PMC6058198

[19] Sakamoto J, Yamashita Y, Morimoto T, ; COMMAND VTE Registry Investigators. Cancer-associated venous thromboembolism in the real world—from the COMMAND VTE Registry. Circ J. 2019;83(11):2271-2281. doi:10.1253/circj.CJ-19-0515 31548438

[20] Berger ML, Sox H, Willke RJ, . Good practices for real-world data studies of treatment and/or comparative effectiveness: recommendations from the joint ISPOR-ISPE Special Task Force on Real-World Evidence in Health Care Decision Making. Value Health. 2017;20(8):1003-1008. doi:10.1016/j.jval.2017.08.3019 28964430

[21] Pasina L, Brucato AL, Falcone C, . Medication non-adherence among elderly patients newly discharged and receiving polypharmacy. Drugs Aging. 2014;31(4):283-289. doi:10.1007/s40266-014-0163-7 24604085

[22] Yao X, Inselman JW, Ross JS, . Comparative effectiveness and safety of oral anticoagulants across kidney function in patients with atrial fibrillation. Circ Cardiovasc Qual Outcomes. 2020;13(10):e006515. doi:10.1161/CIRCOUTCOMES.120.006515 33012172 PMC7580213

[23] McCaffrey DF, Griffin BA, Almirall D, Slaughter ME, Ramchand R, Burgette LF. A tutorial on propensity score estimation for multiple treatments using generalized boosted models. Stat Med. 2013;32(19):3388-3414. doi:10.1002/sim.5753 23508673 PMC3710547

[24] Delate T, Charlu M, Zhu S, . Temporal trends in first-line outpatient anticoagulation treatment for cancer-associated venous thromboembolism. Thromb Res. 2020;196:367-370. doi:10.1016/j.thromres.2020.09.008 32979674

[25] Chen A, Stecker E, Warden BA. Direct oral anticoagulant use: a practical guide to common clinical challenges. J Am Heart Assoc. 2020;9(13):e017559. doi:10.1161/JAHA.120.017559 32538234 PMC7670541

[26] Rose DK, Bar B. Direct oral anticoagulant agents: pharmacologic profile, indications, coagulation monitoring, and reversal agents. J Stroke Cerebrovasc Dis. 2018;27(8):2049-2058. doi:10.1016/j.jstrokecerebrovasdis.2018.04.004 29753603

[27] Chiasakul T, Redd R, Patell R, . Overall survival with warfarin vs low-molecular-weight heparin in cancer-associated thrombosis. J Thromb Haemost. 2021;19(11):2825-2834. doi:10.1111/jth.15519 34490999 PMC8530982

[28] Guo JD, Hlavacek P, Poretta T, . Inpatient and outpatient treatment patterns of cancer-associated thrombosis in the United States. J Thromb Thrombolysis. 2020;50(2):386-394. doi:10.1007/s11239-019-02032-3 31955338 PMC7366581

[29] Papakotoulas P, Tsoukalas N, Christopoulou A, . Management of cancer-associated thrombosis (CAT): symptomatic or incidental. Anticancer Res. 2020;40(1):305-313. doi:10.21873/anticanres.13954 31892581

[30] Cohen A, Keshishian A, Lee T, . Effectiveness and safety of apixaban, low-molecular-weight heparin, and warfarin among venous thromboembolism patients with active cancer: a US claims data analysis. Thromb Haemost. 2021;121(3):383-395. doi:10.1055/s-0040-1718728 33171521 PMC7895542

[31] Foerster KI, Hermann S, Mikus G, Haefeli WE. Drug-drug interactions with direct oral anticoagulants. Clin Pharmacokinet. 2020;59(8):967-980. doi:10.1007/s40262-020-00879-x 32157630 PMC7403169

[32] Johnstone C, Rich SE. Bleeding in cancer patients and its treatment: a review. Ann Palliat Med. 2018;7(2):265-273. doi:10.21037/apm.2017.11.01 29307210

[33] Wu Y, Zhang C, Gu Z-C. Cost-effectiveness analysis of direct oral anticoagulants vs. vitamin k antagonists in the elderly with atrial fibrillation: insights from the evidence in a real-world setting. Front Cardiovasc Med. 2021;8(597):675200. doi:10.3389/fcvm.2021.675200 34268343 PMC8275875

[34] Essien UR, Kim N, Magnani JW, . Association of race and ethnicity and anticoagulation in patients with atrial fibrillation dually enrolled in Veterans Health Administration and Medicare: effects of Medicare Part D on prescribing disparities. Circ Cardiovasc Qual Outcomes. 2022;15(2):e008389. doi:10.1161/CIRCOUTCOMES.121.008389 34779655

[35] Essien UR, Magnani JW, Chen N, Gellad WF, Fine MJ, Hernandez I. Race/ethnicity and sex-related differences in direct oral anticoagulant initiation in newly diagnosed atrial fibrillation: a retrospective study of Medicare data. J Natl Med Assoc. 2020;112(1):103-108. doi:10.1016/j.jnma.2019.10.003 32035755 PMC7183759

[36] Nathan AS, Geng Z, Dayoub EJ, . Racial, ethnic, and socioeconomic inequities in the prescription of direct oral anticoagulants in patients with venous thromboembolism in the United States. Circ Cardiovasc Qual Outcomes. 2019;12(4):e005600. doi:10.1161/CIRCOUTCOMES.119.005600 30950652 PMC9119738

[37] Giugliano RP, Ruff CT, Braunwald E, ; ENGAGE AF-TIMI 48 Investigators. Edoxaban versus warfarin in patients with atrial fibrillation. N Engl J Med. 2013;369(22):2093-2104. doi:10.1056/NEJMoa1310907 24251359

[38] Granger CB, Alexander JH, McMurray JJV, ; ARISTOTLE Committees and Investigators. Apixaban versus warfarin in patients with atrial fibrillation. N Engl J Med. 2011;365(11):981-992. doi:10.1056/NEJMoa1107039 21870978

[39] Patel MR, Mahaffey KW, Garg J, ; ROCKET AF Investigators. Rivaroxaban versus warfarin in nonvalvular atrial fibrillation. N Engl J Med. 2011;365(10):883-891. doi:10.1056/NEJMoa1009638 21830957

[40] Sobieraj DM, Baker WL, Smith E, . Anticoagulation for the treatment of cancer-associated thrombosis: a systematic review and network meta-analysis of randomized trials. Clin Appl Thromb Hemost. 2018;24(9_suppl)(suppl):182S-187S. doi:10.1177/1076029618800792 30244595 PMC6714836

[41] Ueyama H, Miyashita H, Takagi H, . Network meta-analysis of anticoagulation strategies for venous thromboembolism in patients with cancer. J Thromb Thrombolysis. 2021;51(1):102-111. doi:10.1007/s11239-020-02151-2 32458316

[42] Ashrani AA, Gullerud RE, Petterson TM, Marks RS, Bailey KR, Heit JA. Risk factors for incident venous thromboembolism in active cancer patients: a population based case-control study. Thromb Res. 2016;139:29-37. doi:10.1016/j.thromres.2016.01.002 26916293 PMC4769375

[43] Petterson TM, Marks RS, Ashrani AA, Bailey KR, Heit JA. Risk of site-specific cancer in incident venous thromboembolism: a population-based study. Thromb Res. 2015;135(3):472-478. doi:10.1016/j.thromres.2014.12.013 25547213 PMC4339484

[44] Brunetti ND, Tricarico L, Correale M, . Direct oral anticoagulants more effective than low-molecular-weight heparin for venous thrombo-embolism in cancer: an updated meta-analysis of randomized trials. J Thromb Thrombolysis. 2020;50(2):305-310. 31654194 10.1007/s11239-019-01974-y

[45] Li A, Garcia DA, Lyman GH, Carrier M. Direct oral anticoagulant (DOAC) versus low-molecular-weight heparin (LMWH) for treatment of cancer associated thrombosis (CAT): a systematic review and meta-analysis. Thromb Res. 2019;173:158-163. doi:10.1016/j.thromres.2018.02.144 29506866 PMC6119655

[46] Wysokinski WE, Houghton DE, Casanegra AI, . Comparison of apixaban to rivaroxaban and enoxaparin in acute cancer-associated venous thromboembolism. Am J Hematol. 2019;94(11):1185-1192. doi:10.1002/ajh.25604 31378995

