# Supplementary material for: Comparative Effectiveness of Anticoagulants in Patients With Cancer-Associated Thrombosis
Source: JAMA Netw Open. 2023 Jul 24;6(7):e2325283. doi: 10.1001/jamanetworkopen.2023.25283 (PMC10366701; doi:10.1001/jamanetworkopen.2023.25283)
Supplement: Supplement 1. — eMethods. Detailed Methodology eFigure 1. Patient Selection Flowchart eFigure 2. Cumulative Incidence Curves for (A) Major Bleeding; (B) GI Bleeding; (C) Intracranial Bleeding eFigure 3. Reconciliation of Study Characteristics and Results With Previous Clinical Evidence eTable 1. VTE Risk Stratification According to CCS Cancer Categories eTable 2. ICD Codes for Major Bleeding and Different Sites of Bleeding eTable 3. Additional Baseline Sociodemographic and Clinical Characteristics of Patients Included in the Study eTable 4. Factors Without Significant Associations With Utilization of Anticoagulants in Cancer-Associated Thrombosis eTable 5. Sociodemographic and Clinical Characteristics of Patients After Propensity Score Weighting eTable 6. Factors Associated With Utilization of Anticoagulants in Sensitivity Cohort of Patients (Index Date: January 1, 2018, to September 30, 2019) With Cancer-Associated Thrombosis eTable 7. Post Hoc Sensitivity Analysis for Gastrointestinal (GI) Bleeding in Patients Upper GI Malignant Neoplasm [file jamanetwopen-e2325283-s001.pdf]

Supplemental Online Content

Riaz IB, Fuentes H, Deng Y, et al. Comparative effectiveness of anticoagulants in patients with cancer-associated thrombosis. *JAMA Netw Open.* 2023;6(7):e2325283. doi:10.1001/jamanetworkopen.2023.25283

eMethods. Detailed Methodology

eFigure 1. Patient Selection Flowchart

eFigure 2. Cumulative Incidence Curves for (A) Major Bleeding; (B) GI Bleeding; (C) Intracranial Bleeding

eFigure 3. Reconciliation of Study Characteristics and Results With Previous Clinical Evidence

eTable 1. VTE Risk Stratification According to CCS Cancer Categories

eTable 2. *ICD* Codes for Major Bleeding and Different Sites of Bleeding

eTable 3. Additional Baseline Sociodemographic and Clinical Characteristics of Patients Included in the Study

eTable 4. Factors Without Significant Associations With Utilization of Anticoagulants in Cancer-Associated Thrombosis

eTable 5. Sociodemographic and Clinical Characteristics of Patients After Propensity Score Weighting

eTable 6. Factors Associated With Utilization of Anticoagulants in Sensitivity Cohort of Patients (Index Date: January 1, 2018, to September 30, 2019) With Cancer-Associated Thrombosis

eTable 7. Post Hoc Sensitivity Analysis for Gastrointestinal (GI) Bleeding in Patients Upper GI Malignant Neoplasm

This supplemental material has been provided by the authors to give readers additional information about their work.

© 2023 Riaz IB et al. *JAMA Network Open.*

eMethods. Detailed Methodology

De-identified administrative claims data from OptumLabs® Data Warehouse (OLDW) were analyzed to identify patients with active cancer and acute VTE (1/1/2012 – 9/30/2019). This database contains longitudinal health information on enrollees and patients, representing a diverse mixture of ages, ethnicities, and geographical regions across the United States21. Medical and pharmacy claims, laboratory results, and enrollment records for commercial and Medicare Advantage (MA) enrollees were included in this database. The Mayo Clinic Institutional Review Board exempted this study from review due to the analysis of preexisting, de-identified data.

Study population

Adult patients (≥18 years of age) with a primary cancer diagnosis (except skin cancer) during at least one inpatient or two outpatient visits within 6 months before the VTE date were included. Incident VTE was identified using International Classification Disease [ICD] billing codes between January 1st, 2012, and September 30th, 2019 (eFigure 1). The first diagnosis date of VTE was defined as the date of incident diagnosis. The study cohort was limited to patients who filled an anticoagulant prescription within 30 days after the VTE date. Patients were then categorized into one of three groups: (1) DOAC (2) LMWH or (3) Warfarin based on the initial prescription filled. Within each designated group, anticoagulant management was limited to that specific agent without medication cross-over except for the initial 30 days of treatment where LMWH was required for warfarin, dabigatran, or edoxaban management per FDA labels. The first fill date of a specific anticoagulant was defined as the index therapy and treatment date. Patients who crossed over to a different anticoagulant within the first year were excluded from the analysis.

Patients were also excluded from the analysis for any of the following reasons: (a) prior history of VTE; (b) filled prescription for an oral anticoagulant (warfarin and DOAC) less than one year prior to the VTE index date; or (c) less than 1 year of continuous insurance coverage prior to the VTE index date. Patients were required to have at least one year of continuous enrollment in both medical and pharmacy insurance plans prior to the index date to ensure adequate capture of baseline characteristics.

Demographic and clinical variables were defined by the presence of a claim with corresponding diagnosis codes, procedure codes, or anticoagulant prescription fills. Comorbidities were captured using ICD-9 and ICD-10 codes within one year prior to index date, from which the Charlson comorbidity index was calculated. Cancer types were defined based on the AHRQ CCS categories and regrouped by study personnel. VTE risk was defined based on cancer type (eTable 1). Baseline surgery and chemotherapy were captured within 6 months prior to index date.

Race and ethnicity were abstracted as reported in the database. In the OLDW, ethnicity was assigned by an external vendor who used a rule-based system that combines analysis of first names, middle names, surnames, and surname prefixes and suffixes with geographic criteria. Ethnicity values are then assigned into one of five compliance-determined race/ethnicity code values: W (Non-Hispanic White), B (Non-Hispanic Black), H (Hispanic), A (Asian), and U (Unknown)

Follow-up

Follow-up originated at the VTE index date and continued until the end of treatment, defined as: (a) date of index anticoagulant discontinuation; (b) end of enrollment in health insurance plan; (c) one year after VTE index date; (d) end of the study period (September 30th, 2019); or (d) date of patient death.

Outcomes of Interest

The primary efficacy endpoint included any VTE recurrence. Recurrent deep vein thrombosis (DVT) or pulmonary embolism (PE) were defined as a hospitalization or emergency department visit with a primary diagnosis of DVT or PE using ICD-9 or ICD-10 codes. The primary safety endpoint included any episode of major bleeding and sites of bleeding (gastrointestinal [GI], genitourinary [GU], Intra-cranial bleeding).

© 2023 Riaz IB et al. *JAMA Network Open.*

ICD codes for major bleeding and different sites of bleeding used codes from previous published studies (eTable 2). All-cause mortality was identified using the mortality data from OLDW, which is based on the Social Security Death Master File, deceased status from EHR data, death as a reason for disenrollment, and death indicated by inpatient discharge status.

Statistical Analysis

Baseline characteristics of the treatment cohorts were reported as frequencies with percentages for categorical data and means with standard deviations (SD) for continuous variables. Multinomial logistic regression was used to assess predictors of DOAC relative to other anticoagulants (LMWH and warfarin) and presented as odds ratios (OR) and 95% confidence intervals (CI). Kaplan-Meier curves were plotted to assess the differences in time to medication discontinuation among the three groups.

Propensity score (PS) with inverse probability of treatment weighting was used to balance differences in baseline characteristics among the 3 treatment groups. PS was estimated using generalized boosted models, which uses an iterative process with multiple regression trees to capture complex and nonlinear relationships without over-fitting the data. All baseline characteristics listed in Table 1 and Supplemental Table 3 were included in the PS models to derive the PS and the Average Treatment Effect (ATE) weights. Standardized mean difference was used to assess the balance of covariates and a standardized difference less than 0·1 was considered acceptable.

Weighted Cox proportional hazards regression with a robust variance estimator was used to assess outcomes. The event rates per 100 person-years and hazard ratios (HRs) were calculated and the cumulative incidence curves were plotted. The proportional hazards assumption was tested based on Schoenfeld residuals and found to be valid. $P < 0{\cdot}05$ was considered statistically significant for all 2-sided tests. All analyses were conducted using SAS 9·4 (SAS Institute Inc., Cary, NC), R version 4·0·2 (R Foundation) and Stata version 14·1 (StatCorp, College Station, TX).

© 2023 Riaz IB et al. *JAMA Network Open.*

eFigure 1. Patient Selection Flowchart

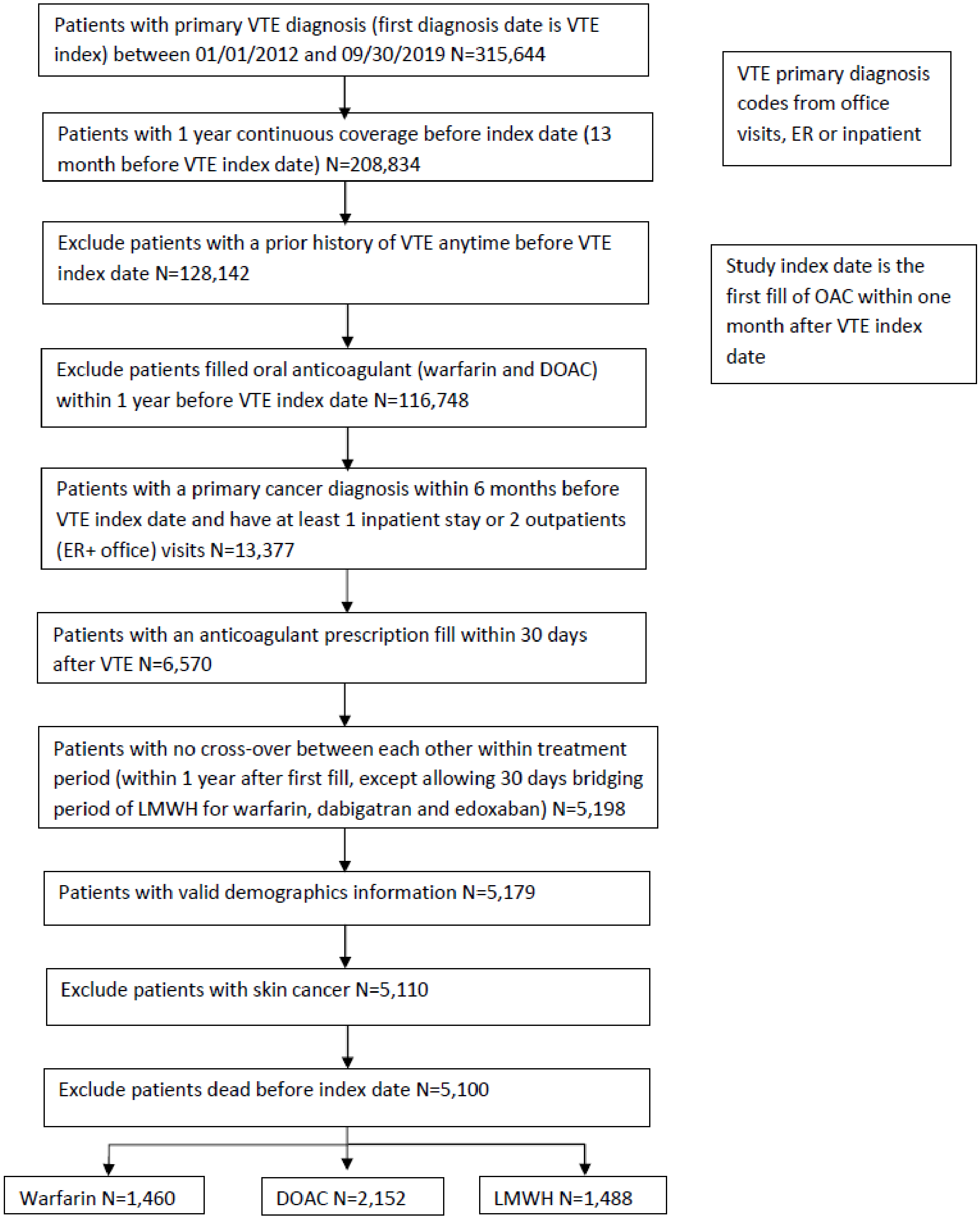

Abbreviations: DOAC: direct oral anticoagulants; LMWH: low molecular weight heparin; VTE: venous thromboembolism, OAC: oral anticoagulants

eFigure 2. Cumulative Incidence Curves for (A) Major Bleeding; (B) GI Bleeding; (C) Intracranial Bleeding

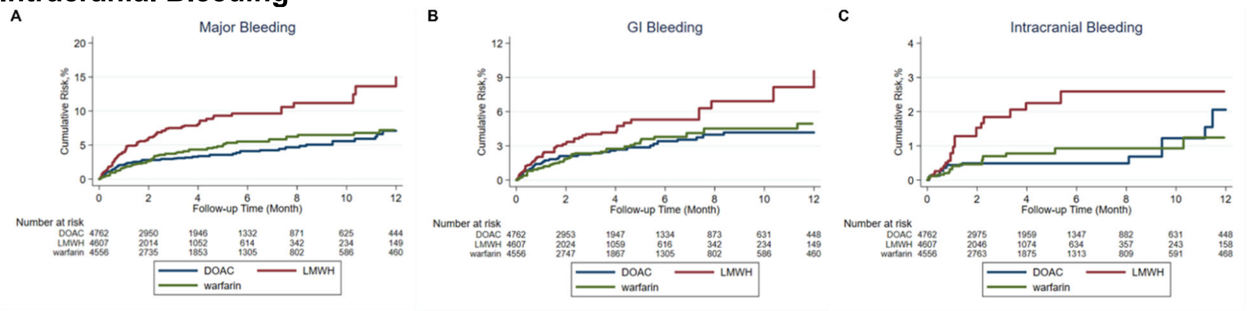

eFigure 3. Reconciliation of Study Characteristics and Results With Previous Clinical Evidence

	Current	Cohen; 2020*<br>PMID: 33171521	Delate D; 2020<br>PMID: 32979674	Guo JD; 2020<br>PMID: 31955338	Papakotoulas P; 2020<br>PMID: 31892581	Sakamoto 2019<br>PMID: 31548438	Khorana AA; 2017<br>PMID: 30046670
**Study design and data sources used**	●	● ● ● ●	● ●	●	■	●	●
**Total number of participants - N**	5100	14086	9816	8125	120	695	2941
**Age**	66.3 (12.3)	64.1 (12.9)	65.8 (12.7)	65.6 (13.0)	63.9 (12.5)	66.5 (12.2)	72.6 (10.1)
**Type of anticoagulation - N (%)**							
**DOACs**	2152 (41.2%)	3393** (24.7%)	188 (1.9%)	730 (8.9%)	NA	20 (2.8%)	709** (24.1%)
**LMWH**	1488 (29.1%)	6108 (43.3%)	3029 (30.9%)	2932 (36%)	120 (100%)	NA	735 (25%)
**Warfarin**	1460 (28.6%)	4585 (32.5%)	6348 (64.7%)	428 (5.8%)	NA	576 (82.8%)	1403 (47.7%)
**Common solid cancers included - N (%)**	Lung: 913 (17.9%)<br>Urologic: 830 (16.3%)<br>Breast: 699 (13.7%)<br>Colorectal: 580 (11.4%)<br>Gynecological: 409 (8.0%)	Solid cancers: 11792 (83.7%)	NSCLC: 1451 (14.8%)<br>Breast: 1265 (12.9%)<br>Colorectal: 1156 (11.8%)<br>Prostate: 806 (8.2%)<br>Pancreas: 550 (5.6%)	Lung: 1500 (18.5%)<br>Breast: 1176 (14.5%)<br>Testis: 724 (8.9%)<br>Colon: 625 (7.7%)<br>Pancreas: 470 (5.8%)	Lung: 24 (20%)<br>Colorectal: 16 (13%)<br>Pancreas: 14 (12%)<br>Breast: 11 (9%)<br>Gastric: 8 (6.5%)	Lung: 114 (16.4%)<br>Colon: 88 (12.7%)<br>Uterine: 61 (8.8%)<br>Ovarian: 59 (8.5%)<br>Gastric: 51 (7.3%)	Lung: 509 (17.3%)<br>Prostate: 326 (11%)<br>Breast: 318 (10.8%)<br>Colorectal: 348 (11.8%)
**Metastatic cancers - N (%)**	3063 (60.1%)	7243 (51.4%)	3796 (38.7%)***	150 (1.8%)	90 (80%)	223 (32%)	NA
**VTE type - N (%)**							
**DVT only**	2405 (47.2%)	8186 (58.1%)	5187 (52.8%)	3743 (46.1%)	56 (51.8%)	302 (43%)	NA
**PE only**	2254 (44.2%)	5900 (41.8%)	1021 (10.4%)	4382 (53.9%)	33 (30.6%)	393 (57%)	NA
**DVT + PE**	441 (8.6%)		3608 (36.8%)	NA	NA		
**Presistence rate at 6 months - %**							
**DOACs**	28.80%	60.6%	NA	NA	NA	45.40%	61%
**LMWH**	13.90%	38.9%	NA	NA	NA		37%
**Warfarin**	30.00%	51.0%	NA	NA	NA		61%
**Outcomes - %**							
**VTE recurrence**	DOACs: 20.6%<br>LMWH: 39.7%<br>Warfarin: 29.9%	DOACs: 15.8%<br>LMWH: 28.8%<br>Warfarin: 22.2%	NA	NA	2.50%	17.70%	NA
**Major bleeding**	DOACs: 9.8%<br>LMWH: 26.7%<br>Warfarin: 11.1%	DOACs: 11.8%<br>LMWH: 20.1%<br>Warfarin: 15.7%	NA	NA	3.30%	26.60%	NA
**GI bleeding**	DOACs: 7.17<br>LMWH: 14.6%<br>Warfarin: 7.38%	DOACs: 3.9%<br>LMWH: 4.3%<br>Warfarin: 4.8%	NA	NA	NA	NA	NA
**Intracranial**	DOACs: 1.84%<br>LMWH: 5.88%<br>Warfarin: 1.93%	DOACs: 0.6%<br>LMWH: 2.9%<br>Warfarin: 1.4%	NA	NA	NA	NA	NA
**All-cause mortality**	DOACs: 11.3%<br>LMWH: 21.1%<br>Warfarin: 13.5%	NA	NA	NA	NA	NA	NA

Retrospective observational design	Prospective observational design
● Optum ClinInformatics Data Mart<br>● IBM MarketScan<br>● Humana Research Database<br>● IQVIA<br>● Kaiser Permanente Northern California (KPNC)<br>● Kaiser Permanente Colorado (KPCO)<br>● COMMAND VTE Registry	■ 18 oncology centers in Greece

Abbreviations: DOAC: direct oral anticoagulants; LMWH: low molecular weight heparin; DVT: deep vein thrombosis; PE: pulmonary embolism.
*Characteristics of Inverse-probability treatment weighted cohorts
**The DOAC in Cohen et al was apixaban, the DOAC in Khorana et al was rivaroxaban.
***Clinical stage 4

© 2023 Riaz IB et al. *JAMA Network Open.*

eTable 1. VTE Risk Stratification According to CCS Cancer Categories

CCS categories	Cancer Type	VTE Risk[a]
Cancer of brain & nervous system	Brain	3
Cancer of breast	Breast	1
Cancer - other & unspecified primary	Cancer of unknown primary	1
Malignant neoplasm without site specification	Cancer of unknown primary	1
Neoplasms of unspecified nature or uncertain behavior	Cancer of unknown primary	1
Cancer of colon	Colorectal	1
Cancer of rectum & anus	Colorectal	1
Cancer of head & neck	ENT	1
Cancer of thyroid	ENT	1
Cancer of other male genital organs	Genitourinary	1
Cancer of other urinary organs	Genitourinary	1
Cancer of ovary	Gynecological	2
Cancer of cervix	Gynecological	1
Cancer of other female genital organs	Gynecological	1
Cancer of uterus	Gynecological	1
Hodgkin disease	Hematological	3
Non-Hodgkin lymphoma	Hematological	3
Leukemias	Hematological	2
Multiple myeloma	Hematological	2
Cancer - other respiratory & intrathoracic	Lung	2
Cancer of bronchus, lung	Lung	2
Cancer of bone & connective tissue	Musculoskeletal	1
Secondary malignancies	Other	1
Cancer of liver & intrahepatic bile duct	Pancreaticobiliary	3
Cancer of pancreas	Pancreaticobiliary	3
Cancer of esophagus	Upper Gastrointestinal	3
Cancer of other GI organs, peritoneum	Upper Gastrointestinal	3
Cancer of stomach	Upper Gastrointestinal	3
Cancer of bladder	Urologic	2
Cancer of kidney & renal pelvis	Urologic	2
Cancer of prostate	Urologic	1
Cancer of testis	Urologic	1

Abbreviations: CCS: clinical classification software; VTE: venous thromboembolism; ENT: ear, nose, and throat.
[a]1: low; 2: intermediate; 3: high

© 2023 Riaz IB et al. *JAMA Network Open.*

eTable 2. ***ICD*** **Codes for Major Bleeding and Different Sites of Bleeding**

Major Bleeding		GI Bleeding		GU Bleeding		Intracranial Bleeding	
**ICD9**	**ICD10**	**ICD9**	**ICD10**	**ICD9**	**ICD10**	**ICD9**	**ICD10**
4230	I60X	5307	I8501	5967	R310	430	I60X
430	I61X	53021	I8511	59971		431	I61X
431	I62X	53082	K2211			432	I62X
432	S0634X	5310	K226			4320	S0634X
4320	S0635X	53100	K250			4321	S0635X
4321	S0636X	53101	K252			4329	S0636X
4329	S0637X	5312	K254			853X	S0637X
4560	S0638X	53120	K256			852X	S0638X
45620	S064X	53121	K260				S064X
4590	S065X	5314	K262				S065X
5307	S066X	53140	K264				S066X
53021	I8501	53141	K266				
53082	I8511	5316	K270				
5310	K2211	53160	K272				
53100	K226	53161	K274				
53101	K250	5320	K276				
5312	K252	53200	K280				
53120	K254	53201	K282				
53121	K256	5322	K284				
5314	K260	53220	K286				
53140	K262	53221	K2901				
53141	K264	5324	K2921				
5316	K266	53240	K2931				
53160	K270	53241	K2941				
53161	K272	5326	K2951				
5320	K274	53260	K2961				
53200	K276	53261	K2971				
53201	K280	5330	K2981				
5322	K282	53300	K2991				
53220	K284	53301	K31811				
53221	K286	5332	K3182				
5324	K2901	53320	K5521				
53240	K2921	53321	K5701				
53241	K2931	5334	K5711				
5326	K2941	53340	K5713				
53260	K2951	53341	K5721				
53261	K2961	5336	K5731				
5330	K2971	53360	K5733				
53300	K2981	53361	K5741				
53301	K2991	5340	K5751				
5332	K31811	53400	K5753				
53320	K3182	53401	K5781				
53321	K5521	5342	K5791				
5334	K5701	53420	K5793				
53340	K5711	53421	K625				
53341	K5713	5344	K6381				
5336	K5721	53440	K920				
53360	K5731	53441	K921				
53361	K5733	5346	K922				
5340	K5741	53460					

© 2023 Riaz IB et al. *JAMA Network Open.*

53400	K5751	53461	
53401	K5753	53501	
5342	K5781	53511	
53420	K5791	53521	
53421	K5793	53531	
5344	K625	53541	
53440	K6381	53551	
53441	K920	53561	
5346	K921	53571	
53460	K922	53783	
53461	I312	53784	
53501	K661	56202	
53511	M250	56203	
53521	R041	56212	
53531	R042	56213	
53541	R310	56881	
53551	R58	5693	
53561		56985	
53571		56986	
53783		578	
53784		5780	
56202		5781	
56203		5789	
56212			
56213			
56881			
5693			
56985			
56986			
578			
5780			
5781			
5789			
5967			
59971			
7191			
71910			
71911			
71912			
71913			
71914			
71915			
71916			
71917			
71918			
71919			
7848			
7863			
853X			
852X			

Abbreviations: GI: gastrointestinal; GU: genitourinary; ICD: International Classification of Diseases

© 2023 Riaz IB et al. *JAMA Network Open.*

eTable 3. Additional Baseline Sociodemographic and Clinical Characteristics of Patients Included in the Study

	DOAC<br>No (%)	LMWH<br>No (%)	Warfarin<br>No (%)	Total<br>No (%)
**Number of Patients**	2152 (100)	1488 (100)	1460 (100)	5100 (100)
**Age**				
Median	69	64	69	68
IQR	60.0, 76.0	56.0, 73.0	61.0, 77.0	59.0, 75.0
**Census Region N (%)**				
South	1082 (50.3%)	586 (39.4%)	550 (37.7%)	2218 (43.5%)
Midwest	560 (26.0%)	432 (29.0%)	486 (33.3%)	1478 (29.0%)
Northeast	264 (12.3%)	281 (18.9%)	224 (15.3%)	769 (15.1%)
West	246 (11.4%)	189 (12.7%)	200 (13.7%)	635 (12.5%)
**VTE hospitalization length**				
N	1263	1003	1090	3356
Mean (SD)	5.3 (5.4)	5.9 (6.5)	6.2 (5.7)	5.8 (5.9)
Median	4	4	5	4
IQR	2.0, 6.0	2.0, 7.0	3.0, 7.0	2.0, 7.0
**Charlson comorbidity index**				
Median	8	9	8	9.0
IQR	4.0, 10.0	8.0, 10.0	4.0, 10.0	4.0, 10.0
**Baseline comorbidities (within 1 year before index date) N (%)**				
Hypertension	1523 (70.8%)	927 (62.3%)	1048 (71.8%)	3498 (68.6%)
Cardiac arrhythmia	841 (39.1%)	570 (38.3%)	540 (37.0%)	1951 (38.3%)
COPD	747 (34.7%)	463 (31.1%)	515 (35.3%)	1725 (33.8%)
Diabetes	627 (29.1%)	412 (27.7%)	432 (29.6%)	1471 (28.8%)
Mild liver disease[a]	527 (24.5%)	478 (32.1%)	336 (23.0%)	1341 (26.3%)
Peripheral vascular disease	480 (22.3%)	244 (16.4%)	317 (21.7%)	1041 (20.4%)
Obesity	400 (18.6%)	239 (16.1%)	239 (16.4%)	878 (17.2%)
Renal disease	383 (17.8%)	193 (13.0%)	295 (20.2%)	871 (17.1%)
Cerebrovascular disease	338 (15.7%)	252 (16.9%)	258 (17.7%)	848 (16.6%)
Congestive heart failure	392 (18.2%)	193 (13.0%)	257 (17.6%)	842 (16.5%)
Chronic kidney disease	306 (14.2%)	135 (9.1%)	223 (15.3%)	664 (13.0%)
Atrial fibrillation	277 (12.9%)	132 (8.9%)	209 (14.3%)	618 (12.1%)
Diabetes with chronic complication	250 (11.6%)	129 (8.7%)	116 (7.9%)	495 (9.7%)
Stroke	170 (7.9%)	123 (8.3%)	112 (7.7%)	405 (7.9%)
Myocardial Infarction	169 (7.9%)	102 (6.9%)	104 (7.1%)	375 (7.4%)
Dementia	73 (3.4%)	49 (3.3%)	105 (7.2%)	227 (4.5%)
Peptic ulcer disease	73 (3.4%)	59 (4.0%)	56 (3.8%)	188 (3.7%)
Hemiplegia or paraplegia	80 (3.7%)	67 (4.5%)	35 (2.4%)	182 (3.6%)
Rheumatic disease	70 (3.3%)	39 (2.6%)	52 (3.6%)	161 (3.2%)
Moderate or severe liver disease[a]	>20[b]	>30[b]	>11[b]	>70[b]
AIDS/HIV	<11[b]	<11[b]	<11	>11[b]

Abbreviations: DOAC: direct oral anticoagulants; LMWH: low molecular weight heparin; IQR: inter-quartile range; COPD: chronic obstructive pulmonary disease; AIDS/HIV: acquired immunodeficiency syndrome/human immunodeficiency virus

[a] Charlson and Elixhauser definition was used to categorize liver disease into mild and moderate or severe disease

[b] N>20, N>30, N<11 was masked to protect patient confidentiality

© 2023 Riaz IB et al. *JAMA Network Open.*

eTable 4. Factors Without Significant Associations With Utilization of Anticoagulants in Cancer-Associated Thrombosis

Variables	Medication [a]	OR	95% CI [b]		P value
**Sociodemographic**					
Age	warfarin	0.994	0.987	1.001	.08
**Gender**					
Female vs. Male	LMWH	0.98	0.827	1.161	.82
Female vs. Male	warfarin	0.938	0.791	1.113	.46
**Race**					
Asian vs. White	LMWH	0.95	0.586	1.54	.83
Asian vs. White	warfarin	0.658	0.389	1.113	.12
Black vs. White	LMWH	0.937	0.762	1.153	.54
Black vs. White	warfarin	1.111	0.912	1.353	.3
Hispanic vs. White	LMWH	1.013	0.777	1.321	.92
Hispanic vs. White	warfarin	0.879	0.665	1.161	.36
**Census Region**					
Northeast vs. Midwest	warfarin	0.921	0.734	1.154	.47
West vs. Midwest	LMWH	1.013	0.793	1.293	.92
West vs. Midwest	warfarin	1.065	0.843	1.346	.6
**Cancer Type**					
Cancer of unknown primary (Yes vs. No)	LMWH	1.796	0.828	3.896	.14
Cancer of unknown primary (Yes vs. No)	warfarin	1.099	0.473	2.552	.83
Musculoskeletal (Yes vs. No)	warfarin	1.948	0.892	4.256	.09
Brain (Yes vs. No)	warfarin	1.178	0.589	2.353	.64
Breast (Yes vs. No)	LMWH	1.688	0.893	3.191	.11
Upper Gastrointestinal (Yes vs. No)	warfarin	1.422	0.745	2.715	.29
ENT (Yes vs. No)	warfarin	1.105	0.537	2.271	.79
Pancreaticobiliary (Yes vs. No)	warfarin	1.269	0.67	2.405	.47
Genitourinary (Yes vs. No)	LMWH	2.312	0.667	8.015	.19
Genitourinary (Yes vs. No)	warfarin	1.033	0.25	4.272	.96
Hematological (Yes vs. No)	LMWH	1.372	0.796	2.362	.25
Hematological (Yes vs. No)	warfarin	1.16	0.659	2.042	.61
Other (Yes vs. No)	LMWH	<0.001	<0.001	>999.999	.93
Other (Yes vs. No)	warfarin	<0.001	<0.001	>999.999	.93
Metastatic solid tumor (Yes vs. No)	LMWH	<0.001	<0.001	>999.999	.84
Metastatic solid tumor (Yes vs. No)	warfarin	<0.001	<0.001	>999.999	.85
**Baseline Interventions**					
Surgery (Yes vs. No)	LMWH	0.96	0.823	1.12	.6
Chemotherapy (Yes vs. No)	warfarin	0.991	0.855	1.15	.91
**VTE Visit Type**					
DVT vs. PE	LMWH	0.984	0.841	1.152	.85
DVT+PE vs. PE	LMWH	1.22	0.94	1.582	.13
DVT+PE vs. PE	warfarin	1.061	0.817	1.377	.66
**VTE Risk**					
2 vs. 1	warfarin	1.282	0.963	1.707	.09
**Charlson Comorbidity Index**					
CCI Score	LMWH	105.139	<0.001	>999.999	.83
CCI Score	warfarin	88.729	<0.001	>999.999	.85
**Baseline Comorbidities**					
Hypertension (Yes vs. No)	LMWH	0.872	0.736	1.033	.11
Hypertension (Yes vs. No)	warfarin	1.071	0.902	1.272	.43
COPD (Yes vs. No)	LMWH	0.009	<0.001	>999.999	.83
COPD (Yes vs. No)	warfarin	0.011	<0.001	>999.999	.85
Diabetes (Yes vs. No)	LMWH	1.585	0.543	4.63	.4
Diabetes (Yes vs. No)	warfarin	0.471	0.097	2.287	.35
Congestive heart failure (Yes vs. No)	LMWH	0.008	<0.001	>999.999	.83

© 2023 Riaz IB et al. *JAMA Network Open.*

Congestive heart failure (Yes vs. No)	warfarin	0.011	<0.001	>999.999	.85
Mild liver disease (Yes vs. No)	LMWH	0.614	0.202	1.871	.39
Mild liver disease (Yes vs. No)	warfarin	0.388	0.092	1.637	.2
Obesity (Yes vs. No)	LMWH	0.833	0.686	1.011	.06
Obesity (Yes vs. No)	warfarin	0.859	0.711	1.039	.12
Cardiac arrhythmia (Yes vs. No)	LMWH	1.056	0.896	1.243	.52
Atrial fibrillation (Yes vs. No)	warfarin	1.182	0.933	1.498	.17
Stroke (Yes vs. No)	LMWH	0.785	0.552	1.116	.18
Stroke (Yes vs. No)	warfarin	0.808	0.574	1.138	.22
Chronic kidney disease (Yes vs. No)	LMWH	0.765	0.539	1.084	.13
Chronic kidney disease (Yes vs. No)	warfarin	0.902	0.66	1.234	.52
Myocardial infarction (Yes vs. No)	LMWH	0.01	<0.001	>999.999	.84
Myocardial infarction (Yes vs. No)	warfarin	0.01	<0.001	>999.999	.84
Peripheral vascular disease (Yes vs. No)	LMWH	0.007	<0.001	>999.999	.82
Peripheral vascular disease (Yes vs. No)	warfarin	0.01	<0.001	>999.999	.84
Cerebrovascular disease (Yes vs. No)	LMWH	0.012	<0.001	>999.999	.84
Cerebrovascular disease (Yes vs. No)	warfarin	0.014	<0.001	>999.999	.8
Dementia (Yes vs. No)	LMWH	0.01	<0.001	>999.999	.83
Dementia (Yes vs. No)	warfarin	0.025	<0.001	>999.999	.87
Peptic ulcer disease (Yes vs. No)	LMWH	0.011	<0.001	>999.999	.84
Peptic ulcer disease (Yes vs. No)	warfarin	0.012	<0.001	>999.999	.85
Diabetes with chronic complication (Yes vs. No)	LMWH	<0.001	<0.001	>999.999	.83
Diabetes with chronic complication (Yes vs. No)	warfarin	<0.001	<0.001	>999.999	.84
Hemiplegia or paraplegia (Yes vs. No)	LMWH	<0.001	<0.001	>999.999	.83
Hemiplegia or paraplegia (Yes vs. No)	warfarin	<0.001	<0.001	>999.999	.84
Renal disease (Yes vs. No)	LMWH	<0.001	<0.001	>999.999	.83
Renal disease (Yes vs. No)	warfarin	<0.001	<0.001	>999.999	.85
Moderate or severe liver disease ((Yes vs. No))	LMWH	<0.001	<0.001	>999.999	.84
Moderate or severe liver disease ((Yes vs. No))	warfarin	<0.001	<0.001	>999.999	.85
AIDS/HIV (Yes vs. No)	LMWH	<0.001	<0.001	>999.999	.82
AIDS/HIV (Yes vs. No)	warfarin	<0.001	<0.001	>999.999	.85
Rheumatic disease (Yes vs. No)	LMWH	0.009	<0.001	>999.999	.83
Rheumatic disease (Yes vs. No)	warfarin	0.013	<0.001	>999.999	.85

Abbreviations: DOAC: direct oral anticoagulants; LMWH: low molecular weight heparin; OR: odds ratio: CI: confidence limits ENT: ear, nose, and throat; VTE: venous thromboembolism; DVT: deep vein thrombosis; PE: pulmonary embolism; ED: emergency department; COPD: chronic obstructive pulmonary disease; AIDS/HIV: acquired immunodeficiency syndrome/human immunodeficiency virus

[a] All comparisons were made against DOACs

[b] Wald 95% confidence limits

© 2023 Riaz IB et al. *JAMA Network Open.*

eTable 5. Sociodemographic and Clinical Characteristics of Patients After Propensity Score Weighting

	DOAC (N[a] = 4762)	LMWH (N[a] = 4607)	Warfarin (N[a] = 4556)	SMD
**Age**				
Mean (SD)	66·6 (11·9)	65·9 (12·4)	66·2 (12·0)	·04
**Gender N (%)**				·01
Female	2508 (52·7%)	2445 (53·1%)	2398 (52·6%)	
Male	2254 (47·3%)	2162 (46·9%)	2158 (47·4%)	
**Race**				·05
White	3331 (69·9%)	3228 (70·1%)	3245 (71·2%)	
Black	747 (15·7%)	696 (15·1%)	737 (16·2%)	
Hispanic	349 (7·3%)	350 (7·6%)	303 (6·6%)	
Asian	105 (2·2%)	106 (2·3%)	84 (1·8%)	
Unknown	231 (4·8%)	228 (4·9%)	188 (4·1%)	
**Census Region**				·05
South	2134(44·8%)	959 (42·5%)	1942 (42·6%)	
Midwest	1375 (28·9%)	1347 (29·2%)	355 (29·7%)	
Northeast	679 (14·3%)	745 (16·2%)	682 (15·0%)	
West	573(12·0%)	557 (12·1%)	577 (12·7%)	
**Cancer Type N (%)**				
Lung	849 (17·8%)	873 (18·9%)	852(18·7%)	·02
Urologic	771 (16·2%)	652 (14·1%)	758 (16·6%)	·05
Breast	690·2 (14·5%)	595·9 (12·9%)	658·8 (14·5%)	·03
Colorectal	526·4 (11·1%)	499·0 (10·8%)	521·5 (11·4%)	·01
Hematological	512·4 (10·8%)	473·4 (10·3%)	459·5 (10·1%)	·02
Gynecological	359·8 (7·6%)	401·9 (8·7%)	369·7 (8·1%)	·03
Pancreaticobiliary	356·9 (7·5%)	403·9 (8·8%)	350·8 (7·7%)	·03
Upper gastrointestinal	230·7 (4·8%)	265·0 (5·8%)	256·0 (5·6%)	·03
Brain	215(4·5%)	227 (4·9%)	182 (4·0%)	·03
ENT	175·6 (3·7%)	183·1 (4·0%)	119·6 (2·6%)	·05
Musculoskeletal	63 (1·3%)	79 (1·7%)	74 (1·6%)	·02
Cancer of unknown primary	72 (1·5%)	69 (1·5%)	45 (1·0%)	·03
Genitourinary	16·7 (0·4%)	27·4 (0·6%)	13·4 (0·3%)	·03
Other	3·9 (0·1%)	0·0 (0·0%)	0·0 (0·0%)	·03
**Surgery within 6 months before index date N (%)**	1787 (37·5%)	1670 (36·3%)	1781 (39·1%)	·04

© 2023 Riaz IB et al. *JAMA Network Open.*

**Baseline chemotherapy within 6 months before index date N (%)**	2601 (54·6%)	2663 (57·8%)	2511 (55·1%)	·04
**VTE Type N (%)**				
DVT	2218·0 (46·6%)	2103·8 (45·7%)	2181·9 (47·9%)	·03
PE	2147·1 (45·1%)	2091·8 (45·4%)	1980·0 (43·5%)	
DVT+PE	397·0 (8·3%)	411·9 (8·9%)	394·4 (8·7%)	
**VTE Visit Type N (%)**				
Hospitalization	3083·5 (64·8%)	3024·4 (65·6%)	3052·3 (67·0%)	·03
ED	1308·0 (27·5%)	1240·9 (26·9%)	1172·4 (25·7%)	
Office	370·5 (7·8%)	342·1 (7·4%)	331·6 (7·3%)	
**VTE Risk N (%)**				
1	2225·3 (46·7%)	2006·6 (43·6%)	2076·6 (45·6%)	·05
2	1571·3 (33·0%)	1571·9 (34·1%)	1530·7 (33·6%)	
3	965·4 (20·3%)	1028·9 (22·3%)	949·1 (20·8%)	
**Charlson comorbidity index**				
Mean (SD)	7·83 (3·64)	8·01 (3·51)	7·78 (3·61)	·04
**Baseline comorbidities (within 1 year before index date)**				
Hypertension	3309·6 (69·5%)	3119·0 (67·7%)	3157·2 (69·3%)	·03
Cardiac arrhythmia	1846·5 (38·8%)	1774·3 (38·5%)	1675·0 (36·8%)	·03
COPD	1610·2 (33·8%)	1551·0 (33·7%)	1566·5 (34·4%)	·01
Diabetes	1358·8 (28·5%)	1333·1 (28·9%)	1294·6 (28·4%)	·01
Mild liver disease	1237·7 (26·0%)	1237·9 (26·9%)	1187·0 (26·1%)	·01
Peripheral vascular disease	984 (20·7%)	870 (18·9%)	921 (20·2%)	·03
Obesity	841·4 (17·7%)	752·6 (16·3%)	784·3 (17·2%)	·02
Cerebrovascular disease	751 (15·8%)	778 (16·9%)	759 (16·7%)	·02
Renal disease [b]	790 (16·6%)	729 (15·8%)	767 (16·8%)	·02
Congestive heart failure	793·8 (16·7%)	681·4 (14·8%)	741·6 (16·3%)	·03
Chronic kidney disease [b]	637 (13·4%)	550 (11·9%)	588 (12·9%)	·03
Atrial fibrillation	584·5 (12·3%)	469·9 (10·2%)	571·2 (12·5%)	·05
Diabetes with chronic complication	469 (9·8%)	442 (9·6%)	387 (8·5%)	·03
Stroke	382 (8·0%)	371 (8·0%)	352 (7·7%)	·01
Myocardial infarction	357(7·5%)	365 (7·9%)	312 (6·9%)	·03
Dementia	189 (4·0%)	170 (3·7%)	201 (4·4%)	·02
Hemiplegia or paraplegia	167 (3·5%)	182 (4·0%)	124 (2·7%)	·05
Peptic ulcer disease	160 (3·4%)	151 (3·3%)	159 (3·5%)	·01
Rheumatic disease	137 (2·9%)	121 (2·6%)	141 (3·1%)	·02
Moderate or severe liver disease	63 (1·3%)	72 (1·6%)	54 (1·2%)	·02
AIDS/HIV	17 (0·4%)	2 (0·0%)	17 (0·4%)	·05

© 2023 Riaz IB et al. *JAMA Network Open.*

Abbreviations: DOAC: direct oral anti-coagulants; LMWH: low molecular weight heparin; VTE: venous thromboembolism, ENT: ear, nose, and throat; DVT: deep vein thrombosis; PE: pulmonary embolism; ED: emergency department; COPD: chronic obstructive pulmonary disease; AID/HIV: acquired immunodeficiency syndrome/ human immunodeficiency virus
[a] Weighted proportion

[b] The definitions are using ICD codes from Charlson and Elixhauser scores. For renal disease, we used ICD9: I12.0, I13.1, N03.2–N03.7, N05.2–N05.7, N18.x, N19.x, N25.0, Z49.0–Z49.2, Z94.0, Z99.2 ICD10: 403.01, 403.11, 403.91, 404.02, 404.03, 404.12, 404.13, 404.92, 404.93, 582.x, 583.0–583.7, 585.x, 586.x, 588.0, V42.0, V45.1, V56.x. For chronic kidney disease, we used ICD9: 403.01, 403.11, 403.91, 404.02, 404.03, 404.12, 404.13, 404.92, 404.93, 585.3, 585.4, 585.5, 585.6, 792.5, 996.81, V42.0, V45.1, V45.11, V45.12, V56, V56.0, V56.1, V56.2, V56.3, V56.31, V56.32, V56.8 ICD10: I12.0, I13.11, I13.2, I95.3, N18.3, N18.4, N18.5, N18.6, R88.0, T86.1, T86.10, T86.11, T86.12, T86.13, T86.19, Y84.1, Z48.22, Z49, Z49.0, Z49.01, Z49.02, Z49.3, Z49.31, Z49.32, Z91.15, Z94.0, Z99.2, T81.502x, T81.512x, T8.1522x, T81.532x, T81.592x, T85.611x, T85.621x, T85.631x, T85.651x, T85.71x

© 2023 Riaz IB et al. *JAMA Network Open.*

eTable 6. Factors Associated With Utilization of Anticoagulants in Sensitivity Cohort of Patients (Index Date: January 1, 2018, to September 30, 2019) With Cancer-Associated Thrombosis

Variables	Medication [a]	OR	95% CI [b]		P value
**Sociodemographic**					
Age	LMWH	0.983	0.971	0.995	0.007
	warfarin	1.011	0.986	1.037	0.4
**Gender**					
Female vs Male	LMWH	0.743	0.541	1.021	0.07
Female vs Male	warfarin	1.138	0.652	1.987	0.65
**Race**					
Asian vs White	LMWH	0.657	0.263	1.638	0.37
Asian vs White	warfarin	0.444	0.055	3.569	0.45
Black vs White	LMWH	0.745	0.497	1.118	0.16
Black vs White	warfarin	0.853	0.444	1.639	0.64
Hispanic vs White	LMWH	0.985	0.614	1.581	0.95
Hispanic vs White	warfarin	0.588	0.215	1.608	0.3
Unknown vs White	LMWH	0.681	0.407	1.141	0.14
Unknown vs White	warfarin	0.802	0.336	1.913	0.62
**Census region**					
Northeast vs Midwest	LMWH	1.428	0.942	2.165	0.09
Northeast vs Midwest	warfarin	0.696	0.325	1.492	0.35
South vs Midwest	LMWH	0.786	0.565	1.093	0.15
South vs Midwest	warfarin	0.613	0.35	1.073	0.09
West vs Midwest	LMWH	1.118	0.713	1.752	0.63
West vs Midwest	warfarin	0.539	0.194	1.497	0.24
**Cancer Type**					
Lung (Yes vs No)	LMWH	6.451	2.206	18.86	0.001
Lung (Yes vs No)	warfarin	3.092	0.425	22.514	0.27
Urologic (Yes vs No)	LMWH	5.117	1.723	15.195	0.003
Urologic (Yes vs No)	warfarin	1.62	0.227	11.587	0.63
Musculoskeletal (Yes vs No)	LMWH	7.251	1.862	28.241	0.004
Musculoskeletal (Yes vs No)	warfarin	0.001	<0.001	>999.999	0.88
Brain (Yes vs No)	LMWH	7.118	2.244	22.58	0.001
Brain (Yes vs No)	warfarin	4.635	0.495	43.401	0.18
Breast (Yes vs No)	LMWH	4.888	1.524	15.679	0.008
Breast (Yes vs No)	warfarin	3.499	0.479	25.554	0.22
Gynecological (Yes vs No)	LMWH	14.046	4.563	43.234	<.0001

© 2023 Riaz IB et al. *JAMA Network Open.*

Gynecological (Yes vs No)	warfarin	1.388	0.155	12.461	0.77
Colorectal (Yes vs No)	LMWH	5.378	1.64	17.64	0.006
Colorectal (Yes vs No)	warfarin	4.152	0.512	33.653	0.18
Upper gastrointestinal (Yes vs No)	LMWH	4.762	1.567	14.477	0.006
Upper gastrointestinal (Yes vs No)	warfarin	2.801	0.274	28.578	0.38
ENT (Yes vs No)	LMWH	5.455	1.561	19.06	0.008
ENT (Yes vs No)	warfarin	0.542	0.033	9.015	0.67
Pancreaticobiliary (Yes vs No)	LMWH	4.146	1.377	12.481	0.01
Pancreaticobiliary (Yes vs No)	warfarin	2.998	0.326	27.583	0.33
Genitourinary (Yes vs No)	LMWH	4.033	0.531	30.625	0.18
Genitourinary (Yes vs No)	warfarin	<0.001	<0.001	>999.999	0.94
Hematological (Yes vs No)	LMWH	2.237	0.846	5.911	0.1
Hematological (Yes vs No)	warfarin	1.033	0.164	6.523	0.97
Other (Yes vs No)	LMWH	0.002	<0.001	>999.999	0.96
Other (Yes vs No)	warfarin	<0.001	<0.001	>999.999	0.98
**VTE type**					
DVT vs PE	LMWH	0.919	0.688	1.227	0.57
DVT vs PE	warfarin	1.466	0.884	2.432	0.14
DVT+PE vs PE	LMWH	1.565	1.011	2.424	0.04
DVT+PE vs PE	warfarin	0.734	0.272	1.982	0.54
**VTE visit type**					
ED vs Hospitalization	LMWH	0.585	0.429	0.799	0.001
ED vs Hospitalization	warfarin	0.501	0.278	0.903	0.02
Office vs hospitalization	LMWH	0.695	0.403	1.198	0.19
Office vs hospitalization	warfarin	0.779	0.299	2.027	0.61
**VTE risk**					
2 vs 1	LMWH	0.988	0.567	1.724	0.97
2 vs 1	warfarin	1.458	0.494	4.303	0.49
3 vs 1	LMWH	3.068	1.114	8.448	0.03
3 vs 1	warfarin	1.293	0.187	8.933	0.79
**Baseline intervention**					
Baseline surgery (Yes vs No)	LMWH	0.713	0.532	0.956	0.02
Baseline surgery (Yes vs No)	warfarin	0.948	0.562	1.599	0.84
Baseline chemotherapy (Yes vs No)	LMWH	1.151	0.87	1.523	0.32
Baseline chemotherapy (Yes vs No)	warfarin	0.774	0.468	1.282	0.32
**Charlson Comorbidity Index**					
Charlson index	LMWH	48.5	<0.001	>999.999	0.87

© 2023 Riaz IB et al. *JAMA Network Open.*

Charlson index	warfarin	67.245	<0.001	>999.999	0.89
Baseline comorbidities					
Hypertension (Yes vs No)	LMWH	0.863	0.623	1.195	0.37
Hypertension (Yes vs No)	warfarin	1.678	0.812	3.467	0.16
COPD (Yes vs No)	LMWH	0.015	<0.001	>999.999	0.86
COPD (Yes vs No)	warfarin	0.009	<0.001	>999.999	0.87
Diabetes (Yes vs No)	LMWH	1.526	0.221	10.53	0.67
Diabetes (Yes vs No)	warfarin	<0.001	<0.001	>999.999	0.91
Congestive heart failure (Yes vs No)	LMWH	0.019	<0.001	>999.999	0.87
Congestive heart failure (Yes vs No)	warfarin	0.015	<0.001	>999.999	0.89
Obesity (Yes vs No)	LMWH	1.242	0.887	1.739	0.21
Obesity (Yes vs No)	warfarin	1.958	1.123	3.412	0.02
Cardiac arrhythmia (Yes vs No)	LMWH	0.966	0.715	1.306	0.82
Cardiac arrhythmia (Yes vs No)	warfarin	0.925	0.529	1.62	0.79
Atrial fibrillation (Yes vs No)	LMWH	0.689	0.427	1.111	0.13
Atrial fibrillation (Yes vs No)	warfarin	1.794	0.914	3.522	0.09
Stroke (Yes vs No)	LMWH	1.085	0.587	2.005	0.8
Stroke (Yes vs No)	warfarin	1.713	0.639	4.594	0.28
Chronic kidney disease (Yes vs No)	LMWH	0.928	0.5	1.723	0.81
Chronic kidney disease (Yes vs No)	warfarin	2.062	0.782	5.437	0.14
Myocardial infarction (Yes vs No)	LMWH	0.02	<0.001	>999.999	0.87
Myocardial infarction (Yes vs No)	warfarin	0.015	<0.001	>999.999	0.89
Peripheral vascular disease (Yes vs No)	LMWH	0.016	<0.001	>999.999	0.86
Peripheral vascular disease (Yes vs No)	warfarin	0.009	<0.001	>999.999	0.87
Cerebrovascular disease (Yes vs No)	LMWH	0.028	<0.001	>999.999	0.88
Cerebrovascular disease (Yes vs No)	warfarin	0.011	<0.001	>999.999	0.88
Dementia (Yes vs No)	LMWH	0.01	<0.001	>999.999	0.84
Dementia (Yes vs No)	warfarin	0.024	<0.001	>999.999	0.9
Peptic ulcer disease (Yes vs No)	LMWH	0.022	<0.001	>999.999	0.87
Peptic ulcer disease (Yes vs No)	warfarin	0.011	<0.001	>999.999	0.88
Mild liver disease (Yes vs No)	LMWH	0.055	<0.001	>999.999	0.9
Mild liver disease (Yes vs No)	warfarin	68.807	<0.001	>999.999	0.94
Diabetes with chronic complication (Yes vs No)	LMWH	<0.001	<0.001	>999.999	0.87
Diabetes with chronic complication (Yes vs No)	warfarin	<0.001	<0.001	>999.999	0.88
Hemiplegia or paraplegia (Yes vs No)	LMWH	<0.001	<0.001	>999.999	0.86
Hemiplegia or paraplegia (Yes vs No)	warfarin	<0.001	<0.001	>999.999	0.88
Renal disease (Yes vs No)	LMWH	<0.001	<0.001	>999.999	0.87

© 2023 Riaz IB et al. *JAMA Network Open.*

Renal disease (Yes vs No)	warfarin	<0.001	<0.001	>999.999	0.89
Moderate or severe liver disease (Yes vs No)	LMWH	<0.001	<0.001	>999.999	0.87
Moderate or severe liver disease (Yes vs No)	warfarin	<0.001	<0.001	>999.999	0.9
Metastatic cancer (Yes vs No)	LMWH	<0.001	<0.001	>999.999	0.87
Metastatic cancer (Yes vs No)	warfarin	<0.001	<0.001	>999.999	0.89
AIDS/HIV (Yes vs No)	LMWH	<0.001	<0.001	>999.999	0.83
AIDS/HIV (Yes vs No)	warfarin	<0.001	<0.001	>999.999	0.89
Rheumatic disease (Yes vs No)	LMWH	0.028	<0.001	>999.999	0.88
Rheumatic disease (Yes vs No)	warfarin	0.02	<0.001	>999.999	0.89

Abbreviations: DOAC: direct oral anticoagulants; LMWH: low molecular weight heparin; OR: odds ratio: CI: confidence limits ENT: ear, nose, and throat; VTE: venous thromboembolism; DVT: deep vein thrombosis; PE: pulmonary embolism; ED: emergency department; COPD: chronic obstructive pulmonary disease; AIDS/HIV: acquired immunodeficiency syndrome/human immunodeficiency virus

[a] All comparisons were made against DOACs

[b] Wald 95% confidence limits

© 2023 Riaz IB et al. *JAMA Network Open.*

eTable 7. Post Hoc Sensitivity Analysis for Gastrointestinal (GI) Bleeding in Patients Upper GI Malignant Neoplasm

Outcomes	No of patients	No. of events	Person-years	Events per 100 person-years	Hazard Ratio (95% CI)	P-value
**GI bleeding**						
LMWH	96	9	58.5	15.56	0.83 (0.21, 3.31)	0.79
Warfarin	93	5	81.6	6.06	0.34 (0.06, 2.04)	0.24
DOAC	84	12	66.93	17.9	Reference	

There is no significant difference in the GI bleeding detected in the subgroup. However, also please note the sample are small in this subpopulation and wide confidence intervals which might have precluded statistical significance.

© 2023 Riaz IB et al. *JAMA Network Open.*
