# Supplementary material for: Comparative Effectiveness of Anticoagulants in Patients With Cancer-Associated Thrombosis
Source: JAMA Netw Open. 2023 Jul 24;6(7):e2325283. doi: 10.1001/jamanetworkopen.2023.25283 (PMC10366701; doi:10.1001/jamanetworkopen.2023.25283)
Supplement: Supplement 2. — Data Sharing Statement [file jamanetwopen-e2325283-s002.pdf]

Data Sharing Statement

Riaz. Comparative Effectiveness of Anticoagulants in Patients With Cancer-Associated Thrombosis. *JAMA Netw Open*. Published July 24, 2023.
doi:10.1001/jamanetworkopen.2023.25283

Data
Data available: No

Additional Information
Explanation for why data not available: Administrative claims-based patient data was used for the analyses
